# Reading Difficulties in Individuals with Homonymous Visual Field Defects: A Systematic Review of Reported Interventions

**DOI:** 10.1007/s11065-024-09636-4

**Published:** 2024-04-19

**Authors:** S. Tol, G. A. de Haan, E. M. J. L. Postuma, J. L. Jansen, J. Heutink

**Affiliations:** 1 Clinical and Developmental Neuropsychology, University of Groningen, Grote Kruisstraat 2/1, 9712 TS Groningen, The Netherlands; 2 Royal Dutch Visio, Centre of Expertise for Blind and Partially Sighted People, Amersfoortsestraatweg 180, 1272 RR Huizen, The Netherlands

**Keywords:** Homonymous visual field defects, Hemianopia, Intervention, Reading

## Abstract

Reading difficulties are amongst the most commonly reported problems in individuals with homonymous visual field defects (HVFDs). To be able to provide guidance for healthcare professionals considering offering reading training, researchers in this field and interested individuals with HVFDs, this systematic review aims to (1) provide an overview of the contextual and intervention characteristics of all published HVFD interventions and (2) generate insights into the different reading outcome measures that these studies adopted. A search on PsycINFO, MEDLINE and Web of Science was conducted up to February 2, 2023. All intervention studies for HVFD in which reading was measured were included. Data was collected about the intervention type, session duration, number of sessions, the intensity, duration, circumstance of the interventions, country in which the intervention was studied and reading measures. Sixty records are included, describing 70 interventions in total of which 21 are specifically reading interventions. Overall, adjusted saccadic behaviour interventions occur most in the literature. A wide range within all intervention characteristics was observed. Forty-nine records reported task-performance reading measures, and 33 records reported self-reported reading measures. The majority of task-performance measures are based on self-developed paragraph reading tasks with a time-based outcome measure (e.g. words per minute). Future research could benefit from making use of validated reading tests, approaching the measurement of reading mixed-methods and providing participants the possibility to supply outcomes relevant to them.

## Introduction

Homonymous visual field defects (HVFD) are a common consequence of acquired brain injury, such as stroke (Gilhotra et al., 2002; Zhang et al., 2006). A particularly bothersome effect of HVFDs can be problems in the ability to read. Reading difficulties are reported in up to 80% of the individuals with HVFDs (iwH; de Haan et al., 2015a, b; Zihl, 2011) and can have a severe impact on daily life (Lane et al., 2008; Schuett, 2009). The location of the neurological damage determines the side and size of the visual field defect (Siegel & Sapru, 2015; Zihl, 2011), which in turn may influence the nature and amount of reading difficulties (e.g. Schuett, 2009; Trauzettel-Klosinski, 2010; Trauzettel-Klosinski & Brendler, 1998; Zihl, 2011). Therefore, the nature and severity of reading difficulties vary between iwHs. Reported reading difficulties by iwHs include reduced reading speed, missing words in the affected visual field, trouble finding the beginning of the line, losing track of the line, lacking overview and being “stuck” at a word (e.g. Bergsma & van der Wildt, 2010; Poggel et al., 2010; Schuett et al., 2008). The described difficulties have a negative influence on text comprehension, the amount of time individuals are able to read continuously and the degree to which iwHs are able to memorise the information (Schuett, 2009; Zihl, 2011).

In deciding upon the best treatment option, clinicians have to integrate the client’s personal circumstances and desires (e.g. motivation, special demands, severity of disease, cognitive abilities), as well as their own clinical expertise and the best available scientific evidence, according to evidence-based medicine (Sackett et al., 1996). For finding the best available evidence, multiple systematic reviews and meta-analyses have provided useful information about current empirically supported HVFD interventions on reading, see e.g. Liu et al. (2019), Maeyama et al. (2023) and Pollock et al. (2019). What these reviews all point out however is a well-established lack of high-quality HVFD intervention studies for the effectiveness of HVFD interventions on reading. Additionally, proven efficacy or effectiveness of an intervention in a group of people with reading difficulties due to HVFDs is no guarantee that it will be successful for any individual, let alone that the intervention matches important factors such as their personal needs, limitations and circumstances (Falkenberg et al., 2020; Skivington et al., 2021). Thus, no robust guidelines for the treatment of HVFD-induced reading difficulties exist. Taking into account the preferred integration of individual differences in personal and clinical circumstances, it is therefore important that clinicians are aware of the different characteristics of existing interventions. This includes interventions for which effectiveness or efficacy may not have been established by means of high-quality study designs, but which do have sound theoretical underpinnings or are at least in some aspects similar to interventions that have proven to be effective. Randomised Controlled Trials (RCTs) are considered the best method to establish an intended effect of intervention (Bosdriesz et al., 2020), and RCTs or similar clinical trial designs are therefore usually the primary focus in HVFD intervention reviews. However, all study designs have strengths and weaknesses, and it has been advised to explore different study designs in complex intervention research considering the research question and context at hand (Bosdriesz et al., 2020; Skivington et al., 2021). For example, RCTs on HVFD reading interventions can give the most relevant information for improving reading performance in iwHs. Concurrently, observational study designs provide a better opportunity to include more representative and therefore generalizable samples, as well as the opportunity to find unintended effects of an intervention (Bosdriesz et al., 2020). To the best of our knowledge, no overview exists of *all* published HVFD interventions in which a reading outcome measure is reported.

The first aim of the current study is to provide a comprehensive overview of all published HVFD interventions in which reading outcome measures were included. We intend to provide this overview not by focussing on the effectiveness of these interventions, but instead by providing a comprehensive overview of intervention and sample characteristics. The outcome of this systematic review can inform clinicians, researchers and interested iwHs about the spectrum of published HVFD interventions. The information provided by this review, taken together with the clinical setting and personal circumstances of the iwH, can be used for finding an appropriate intervention for the individual case.

Kersting and colleagues (2020) argue that individuals in need of care need to be properly informed about intervention outcomes to be able to make informed and personally relevant decisions in a shared decision-making context. Next to locating different treatment options, it is essential to know what outcome measures are relevant for individuals in need of care, and whether these measures are actually used in the intervention research relevant for them. Improvement on reading performance can be objectified as an improvement in reading speed or a decrease in reading errors (e.g. de Jong et al., 2016; Schuett & Zihl, 2013). However, the experienced value of improved reading speed or making less reading errors may differ between iwHs and guide their treatment goals accordingly. While one iwH might experience problems due to not being able to keep up with university-level reading anymore, another iwH might want to be able to read personal emails again. According to Lane et al. (2008), the transfer of intervention outcome to daily life activities has been receiving insufficient attention in HFVD intervention research. Additionally, Schuett (2009) touched upon the importance of the development and use of reading outcome measures to increase comparability between HVFD intervention studies.

Therefore, the second aim of the current study is to generate insights into the different reading outcome measures that HVFD intervention studies have adopted. In this way, the current review can be used to relate available intervention research to individually relevant reading outcome measures. HVFD researchers can consider the outcomes of this review when designing HVFD (reading) intervention studies and in selecting reading outcome measures.

## Method

The protocol for this systematic review was published in the PROSPERO database on July 16, 2020, under the registration code CRD42020192424 (Tol et al., 2020). In light of transparency, we would like to report the aspects of this protocol that do not correspond with the methods of the current review. First, sub-questions about the relationship between the outcome measures and reported reading problems and the aim of the intervention (for reading or for other reasons) are not explicitly addressed in the current review. Secondly, intervention material was not included in the data extraction on intervention characteristics, whereas the country of data collection was included. Lastly, a different quality assessment tool was chosen for the current review. Argumentation for the method of the current review is provided in the method section. The Preferred Reporting Items for Systematic review and Meta-Analysis Protocol (PRISMA-P) was used as guidance for conducting and reporting the current review (Moher et al., 2009). We did not include a meta-analysis in this study because our major goal is to present all HVFD intervention studies with a reading outcome measure in order to provide an overview of the intervention characteristics and reading measures. Conducting a meta-analysis requires the meaningful inclusion of similar study designs and similar outcome measures (Borenstein et al., 2009), which would result in excluding certain HVFD intervention studies. Furthermore, as mentioned, it is not the goal of this review to add to the already existing reviews on HVFD interventions and their effectiveness on reading.

### Eligibility Criteria

For records to be included in the current review, the following criteria were applied: (1) interventions designed for iwHs due to post-chiasmal acquired brain injury, (2) inclusion of iwH in the study and report of their patient characteristics (*n* = 1 studies also eligible) and (3) at least one effect outcome measure had to be included reflecting reading performance, reading experience or reading-related quality of life. Not only interventions specifically designed to improve reading for iwH but rather interventions specifically designed for iwH, in general, were accepted, as long as reading was amongst the outcome measures. Records were excluded if they (1) were not published in either English, German or Dutch language or (2) were not published in a peer-reviewed journal. Records reporting on iwH with comorbid neglect were excluded unless there was a subsample of iwH without neglect described in the paper.

### Search Strategy

The search for records was performed in the databases PsycINFO, MEDLINE and Web of Science up to the date of February 2, 2023. The search terms comprised synonyms of the three keywords ‘hemianopia’, ‘reading’ and ‘intervention’ (Table 1).

**Table 1:** Search string

	Search terms
First term	
*MEDLINE, PsycINFO*	Hemianopia OR hemianopsia OR hemiopia OR hemianopic OR visual field disorder* OR visual field loss OR visual field defect* OR homonymous field defect OR quadrantanopia OR quadrantanopsia OR cerebral blindness OR cortical blindness OR scotoma OR Hemiamblyopia
*Web of Science*	Hemianopia OR hemianopsia OR hemiopia OR hemianopic OR visual “Near” field “Near” disorder* OR visual “Near” field “Near” loss OR visual “Near” field “Near” defect* OR homonymous “Near” field “Near” defect OR quadrantanopia OR quadrantanopsia OR cerebral “Near” blindness OR cortical “Near” blindness OR scotoma OR Hemiamblyopia
AND	
Second term	Reading OR alexia OR dyslexia
AND	
Third term	Intervention* OR training OR treatment* OR rehabilitation OR exercise* OR strateg*

An asterisk (*) at the end of a term indicates that all possible suffixes were included

### Record Selection

After removing duplicates, the titles and abstracts of the records were screened for inclusion by one evaluator (ST). A second evaluator subsequently screened all excluded records to ensure no relevant records were excluded (JJ for records published before January 2020; EP for records published between January 2020 and February 2023). Consensus between the two evaluators was reached by discussing any remaining uncertain cases. A screening with the same procedure was repeated on the full texts of the remaining records. The reference lists of the included records were checked for additional eligible papers.

### Other Sources

To find eligible publications that might not have come up in the systematic search, we additionally searched for trial protocols leading to potential eligible publications in three trial registers and Google Scholar. These trial registers were ClinicalTrials.gov (https://clinicaltrials.gov; 2 searches: ‘hemianopia homonymous’ and ‘visual field defect’), ISRCTN Registry (www.isrctn.org; 2 searches: ‘hemianopia’ and ‘visual field defect’) and the National Eye Institute Clinical Studies Database (https://clinicalstudies.info.nih.gov/indexpage.aspx; 2 searches: ‘hemianopia’ and ‘visual field defect’). In case a trial protocol was identified as potentially leading to a scientific publication eligible for inclusion in this review and which was not already found in our systematic search, we contacted the principal investigators asking for published records from the related trials. Additionally, we used the search string ‘hemianopia intervention’ on Google Scholar (https://scholar.google.com/). The first 150 results were screened.

### Data Collection and Synthesis

Data was extracted from the included papers by authors ST and EP. Records were divided into interventions explicitly aimed at improving reading (reading interventions: RI) and other interventions for which the effect on reading was measured (other interventions: OI). To provide a comprehensive categorisation of the interventions, we did not assign the interventions to one of the three well-known categories (compensation, restoration and substitution) only. Rather, we focussed on the stated mechanisms of the included interventions and created intervention categories based on these mechanisms. Additionally, data was collected about session duration, number of sessions and the intensity, duration and circumstance of the interventions. The country in which the intervention took place was also extracted as this can provide important information about the geographical context and thus e.g. the developmental possibilities of healthcare interventions (Burchett et al., 2013; O’Cathain et al., 2019). It was extracted which study design was used. Regarding study participants, data was extracted on the sample size, size of the visual field defect (without considering macular sparing, which was not mentioned in most studies), side of the visual field defect and time since lesion. Data was further collected on reading outcome measures and measurement methods. A distinction was made between *task performance* measurements and *self-report* measurements. Measures were categorised as task performance measures if a behavioural aspect of reading was recorded, e.g. a reading task where reading speed was measured. On the contrary, measures were categorised as self-report if the subjective experience or evaluation of the participant was the main outcome of the measure, e.g. a questionnaire or interview. Collated information was tabulated, and a narrative synthesis is provided on this information. Information is presented separately for RI and OI studies (with the exception of information about the country of data collection and reading outcome measures).

### Quality Assessment

The quality of the studies was assessed by two evaluators (ST and EP) independently by means of the Appraisal tool for Cross-Sectional Studies (AXIS; Downes et al., 2016). The AXIS has initially been developed for the quality assessment for cross-sectional studies, whereas the current study accepted a range of study methods, such as RCTs, cohort studies and case studies. Nevertheless, the AXIS was deemed most appropriate with regard to the aim of this review, i.e. to give an overview of the types and quality of studies on interventions for reading difficulties in iwH and not to primarily assess the effects of these interventions. The AXIS tool registers both the quality of design as well as the quality of reporting. For the purpose of this review, scoring of certain items (items 3–17) has been done only by paying attention to the reading-related aspect of the item. A percentage score of the positively evaluated items out of the total of 20 items was computed for every record by calculating the percentage of all items that were given a score of ‘1’ (meaning the aspect mentioned in the item was observed in the record). For items 13 and 19, the scores were reversed from 0 to 1 or 1 to 0 to assure that a score of 1 also refers to a positively evaluated item. Interrater reliability for the quality assessment was calculated as follows:

$$\text{total items in agreement} / \text{total items rated }\times 100$$

## Results

### Included Records

**Fig. 1:**
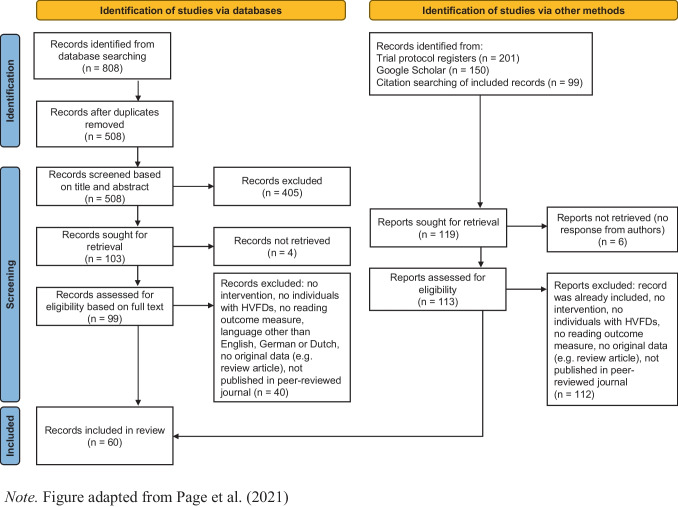
PRISMA 2020 flow diagram of search. Note: Figure adapted from Page et al. (2021)

A flow diagram of the systematic literature search can be found in Fig. 1. After removing duplicates, 508 records were screened, and 60 subsequently met the inclusion criteria. Included records were published between 1985 and 2021. One additional record was included through the Google Scholar search. The following study types were included: 24 pre-post studies (including some with multiple baseline or follow-up measures), 11 RCTs, nine case reports (series), seven case-controlled studies, four controlled trials, two observational studies (defined as data being collected during the intervention), one cross-over study, one prospective non-controlled trial and one prospective cohort study (Table 2). Due to employing identical protocols for both the overview of interventions and for the reading outcome measures, one record was excluded, resulting in a total of 58 records in both cases. The record by Plow et al. (2012a) was excluded from the intervention overviews due to studying the exact same intervention in the same sample as described in the record by Plow et al. (2012b). The record of de Haan et al. (2016) was excluded from the reading outcome measures overview due to the use of the exact same intervention and sample as de Haan et al. (2015b).

**Table 2:** References, study type, quality assessment score, sample characteristics and reported intervention characteristics of the included records

1st author, year [reference number^a^]	Study type	Quality score	*N*	Sample VFD size: *n*	Sample VFD side: *n*	Time since lesion in months: M (SD), min–max	Intervention type	Session duration in minutes	Number of sessions	Intervention intensity	Intervention duration	Intervention circumstance
**Reading interventions**												
Zihl, 2021 [1]	*N*-of-1 case series		97	HH: 97^b^	Left: 52 Right: 44	6(4), 1–24.5	Saccadic adaptation	45	G1: 11.2 on; average G2: 11.5 on average G3: 12.3 on average	1–3 sessions a day	/	Supervised
Hazelton, 2020 [2]	*N*-of-1 case series	80	11^c^	HH: 5 QA: 5 Sectoral: 1	Left: 5 Right: 6	9 median, 6–24	Adjusted saccadic behaviour	10	12	6 days a week	2 weeks	Home
Pineda-Ortiz, 2018 [3]	Case report	55	1	HH: 1	Left: 1	Unclear	Adjusted saccadic behaviour	180	/	Weekly	6 months + 3 months after a 3-month break	/
Aimola, 2014 [4]	RCT	80	28	HH: 20 QA: 8	Left: 15 Right: 13	3–376	Adjusted saccadic behaviour	60	/	Daily	5 weeks (suggested, depended on participant)	Home
Schuett, 2013 [5]	Pre-post study with multiple baseline and FU	75	38^d,e^	HH: 28 QA: 10	Left: 19 Right: 19	G1: 5(3), 1.5–13 G2: 4(3), 1–14	Adjusted saccadic behaviour	45	9 on average	On average 2–2.7 sessions per week	2–3 weeks	Supervised
Ong, 2012 [6]	Pre-post study	85	33	Extent of visual field defect in degrees presented in reference	Right: 33	78, 0–842	Adjusted saccadic behaviour	20 (suggested, depended on participant)	/	Daily (suggested, depended on participant)	20 h	Home
Schuett, 2012 [7]	Controlled trial, cross-over	75	36^d,f^	HH: 25 QA: 5 Other: 6	Left:16 Right: 20	G1: 7(4), 1.5–14 G2: 5(5), 1–18.5	Adjusted saccadic behaviour	45	12 on average	4–6 session per week	2–3 weeks	Supervised
Kerkhoff, 2009 [8]	Observational study	10	50	HH: 50	Left: 25 Right: 25	4	Adjusted saccadic behaviour	40	25	/	/	/
Schuett, 2008 [9]	Pre-post study with multiple baseline	75	20	HH: 12 QA: 4 Paracentral scotoma: 4	Left: 8 Right: 12	7(7), 1–24	Adjusted saccadic behaviour	45	11 on average	On average 5,5 sessions per week	2 weeks	Supervised
			20	HH: 12 QA: 4 Paracentral scotoma: 4	Left: 8 Right: 12	7(12), 1–55	Adjusted saccadic behaviour	45	10 on average	On average 5 sessions per week	2 weeks	Supervised
Spitzyna, 2007 [10]	Controlled trial	70	19^g^	HH: 14 QA: 4 Homonymous scotoma: 1	Right: 19	90, 12–144	Adjusted saccadic behaviour	20	20	On average 5 sessions per week	2 × 4 weeks	Home
Ciuffreda, 2006 [11]	Cross-over	80	2^h^	HH: 2	Right: 2	12	Adjusted saccadic behaviour^i^	60 (36 actual training)	16	2 sessions per week	4 + 4 weeks	Lab
Zihl, 1995 [12]	Pre-post study	55	20	HH: 20	Left: 10 Right: 10	3 weeks, 3–5 weeks	Adjusted saccadic behaviour	/	11 (LH), 22 (RH) on average	2 sessions per day	1–2 weeks	/
Kerkhoff, 1992 [13]	Pre-post study with multiple baseline and FU	55	56	HH: 37 QA: 6 Hemiamblyopia: 9 Paracentral scotoma: 4	Left: 34 Right: 9 Bilateral/diffuse: 13	40, 3–220	Adjusted saccadic behaviour	40	14	Daily, 5 days per week	4–6 weeks	Supervised
Zihl, 1988^j^ [14]	Observational study	40	80	HH: 80	Left: 43 Right: 37	Unclear	Adjusted saccadic behaviour	30–45	/	Usually daily, at least 3 times per week	/	/
Kuester-Gruber, 2020 [15]	RCT	95	21^k^	HH:20 QA:1	Left:11 Right: 10	At least 6	Utilization intact visual field	30	40	2 sessions a day, 5 days a week	4 weeks	Home
de Jong, 2016 [16]	Case controlled	65	13^d^	HH: 9 QA: 4	Left: 7 Right: 6	G1: 33, 12–55 G2: 30, 7–73	Utilization intact visual field	/	/	/	1 session	Lab
Mohamad, 2021 [17]	Case report	65	1	HH: 1	Right: 1	5	Multiple oral re-reading combined with NIBS	60	7	/	6 weeks	/
Lacey, 2015 [18]	Case report	55	1	QA: 1	Right: 1	96	Multiple oral re-reading combined with NIBS	60	5	Daily	5 days	/
Mena-Garcia, 2021 [19]	Case-controlled	85	20	HH: 14 QA: 6	Left: 9 Right: 11	19(28)	Multidisciplinary visual rehabilitation, including reading exercises	30–40	120–168 home-based sessions and 4 office visits	2 sessions a day, 5–7 days/week	12 weeks	4 in-office, rest home-based
Woodhead, 2013 [20]	Pre-post study with multiple baseline and FU	70	8^l^	HH: 3 QA: 5	Right: 8	77, 6–114	Audiovisual word recognition training	20 at least	42	Daily	6 weeks	Self-administered
**Other interventions**												
Zihl, 2021 [1]	*N*-of-1 case series		97	HH: 97^b^	Left: 52 Right: 44	6(4), 1–24.5	Saccadic adaptation	45	G1: 11.2 on average G2: 10.7 on average G3: 12.1 on average	1–3 sessions a day	/	Supervised
Hazelton, 2020 [2]	*N*-of-1 case series	80	11^c^	HH: 5 QA: 5 Other: 1	Left: 5 Right: 6	9 median, 6–24	Adjusted saccadic behaviour	35	8	/	2 weeks	Home
			11^c^	HH: 5 QA: 5 Other: 1	Left: 5 Right: 6	9 median, 6–24	Adjusted saccadic behaviour	45	8	/	5 weeks	Home
			11^c^	HH: 5 QA: 5 Other: 1	Left: 5 Right: 6	9 median, 6–24	Adjusted saccadic behaviour	30	20	2 times a day, 5 days a week	/	Home
Crotty, 2018 [21]	RCT, single-blind	95	13	HH: 8 iHH: 4	Left: 5 Right: 7	47(38)	Adjusted saccadic behaviour	/	9 + 12 sessions	/	3 + 4 weeks	/
Rowe, 2017^ m^ [22]	RCT	80	30	HH: 16 iHH: 14	Left: 13 Right: 17	74(49), 13–172	Adjusted saccadic behaviour	30	42	Daily	6 weeks minimum	/
de Haan, 2016^n^ [23]	Case-controlled	90	45	HH: 35 QA: 10	Left: 31 Right: 14	Unclear	Adjusted saccadic behaviour	60–90	15	/	10 weeks	Clinic
Levy-Bencheton, 2016 [24]	Pre-post study, multiple measurements	75	14	HH: 14	Left: 6 Right: 8	73, 7–180	Adjusted saccadic behaviour	15	3	Every 4–5 weeks	14–21 weeks	Lab
De Haan, 2015b^n^ [25]	RCT	90	26	HH: 21 QA: 5	Left: 18 Right: 8	18(22.5)	Adjusted saccadic behaviour	60–90	15	/	10 weeks	Clinic
Larson, 2015 [26]	Case report	40	1	HH: right eye QA: left eye	Left: 1	1	Adjusted saccadic behaviour	/	24	Weekly	/	In-office
Jacquin-Courtois, 2013 [27]	Case–control	70	7	HH: 5 QA: 2	Left: 3 Right: 4	33(29), 12–96	Adjusted saccadic behaviour	30	1	Once	1 day	Hospital
Schuett, 2013 [5]	Pre-post study with multiple baseline and FU	75	38^d,e^	HH: 28 QA: 10	Left: 19 Right: 19	G1: 5(3), 1.5–13 G2: 4(3), 1–14	Adjusted saccadic behaviour	45	10 on average	/	2 weeks	/
Hayes, 2012 [28]	Case series	60	10	HH: 6 iHH: 4	Left: 4 Right: 6	2 weeks–6 months	Adjusted saccadic behaviour	60	21	3 times per week	7 weeks	Hospital Outside
Mödden, 2012 [29]	RCT	75	15	HH: 10 QA: 5	Unclear	1	Adjusted saccadic behaviour	30	15	/	3 weeks	Supervised
Schuett, 2012 [7]	Controlled trial, cross-over	75	36^d,f^	HH:25 QA:5 Paracentral scotoma:6	Left: 16 Right: 20	G1: 7(4), 1.5–14 G2: 5(5), 1–18.5	Adjusted saccadic behaviour	45	12 on average	/	2–3 weeks	/
Lane, 2010 [30]	Controlled trial	70	42^d,^^p ^	HH: 41 Other: 1	Left: 30 Right: 11 Bilateral: 1	G1: 25(60.5) G2: 14(19)	Adjusted saccadic behaviour	40	15	/	4 weeks on average	Home
Roth, 2009 [31]	RCT	65	15	HH: 12 QA: 3	Unclear	39(55), 1–228	Adjusted saccadic behaviour	30	60	Twice a day, 5 days a week	6 weeks	Home
Bolognini, 2005 [32]	Pre-post study with multiple baseline and FU	55	8^d^	HH: 7 QA: 1	Left: 4 Right: 4	G1: 10, 4–13 G2: 14, 7–24	Adjusted saccadic behaviour	240	14	Daily	2 × 2 weeks	Supervised
Nelles, 2001 [33]	Case-controlled	40	21	HH: 21	Left: 8 Right: 13	1.5, 0.5–24	Adjusted saccadic behaviour	30	8	Twice a week, 2 times a day	4 weeks	Lab
Vasiliou, 1990 [34]	Case report	15	1	HH: 1	Left: 1	60	Adjusted saccadic behaviour	/	/	/	/	Supervised
Zihl, 1985 [35]	Case-controlled	55	55	HH: 44 QA: 11	Unclear	1–28	Adjusted saccadic behaviour	/	/	Daily or 3 times a week	/	/
Bergsma, 2017 [36]	Pre-post study	70	24	HH: 16 iHH: 1 QA: 4 (Paracentral) scotoma: 3	L: 14 R: 9 Upper altitudinal: 1	17 in subacute phase, 7 in chronic phase	Stimulation of impaired visual field	60	5–6 sessions a day	5 days a week	10 weeks at least	Home
Elshout, 2016 [37]	RCT, cross-over	75	27	HH: 20 iHH: 2 QA: 3 Scotoma: 2	Left: 12 Right: 11 Bilateral: 1	26, 0–111	Stimulation of impaired visual field	12 on average	60 min (5 sessions) a day	5 days a week	8 weeks	Home
Bergsma, 2014 [38]	Pre-post study	75	12	HH: 1 iHH: 3 QA: 8	Left: 10 Right: 2	33, 8–105	Stimulation of impaired visual field	60	65	5 days a week	13 weeks	Home
Bergsma, 2012 [39]	Pre-post study	50	12	HH: 6 iHH:3 QA: 2 Scotoma: 1	Left: 10 Right: 2	23(27), 0.5–102	Stimulation of impaired visual field	60	40	/	10 weeks	Supervised
Gall, 2012 [40]	Prospective noncontrolled trial	65	11	HH: 7 iHH: 1 QA: 3	Left: 8 Right: 3	17(15), 1–58	Stimulation of impaired visual field^q^	30	300 or 150	Two times per day, 6 days a week	6 or 3 months	Home
Mödden, 2012^o^ [29]	RCT	75	15	HH: 12 QA: 3	Unclear	1	Stimulation of impaired visual field	30	15	/	3 weeks	Supervised
Bergsma, 2010 [41]	Pre-post study	50	11	HH: 6 iHH: 1 QA: 4	Left: 6 Right: 5	36, 0.5–102	Stimulation of impaired visual field	60	40	Daily	10 weeks	Supervised
Keller, 2010^r^ [42]	Controlled trial	80	10	HH: 6 QA: 4	Left: 7 Right: 3	1, 0.5–2	Stimulation of impaired visual field	30	20	/	3 weeks	/
Poggel, 2010 [43]	Pre-post study	70	19	Size of the area of residual vision presented in reference	Left: 10 Right: 9	36, 7–190	Stimulation of impaired visual field^q^	30–35	56	2 sessions a day	1 month	Home
Jobke, 2009 [44]	RCT, cross-over	60	18^s^	HH: 4 iHH: 3 QA: 2 iQA: 3 Other: 6	Left: 7 Right: 5 Other: 6	94, 40–236	Stimulation of impaired visual field	30	/	Daily	90 days	/
Gall, 2008 [45]	Pre-post study	75	85	HH: 17 iHH: 35 QA: 10 Other: 23	Left: 35 Right: 26 Other: 24	33 (46)	Stimulation of impaired visual field^q^	30	156	Daily, 6 days a week	6 months^s^	Clinic
Poggel, 2008 [46]	Pre-post study	70	19	Size of the area of residual vision presented in reference	Left: 10 Right: 9	36, 7–190	Stimulation of impaired visual field^q^	30–35	56	Twice a day	1 month	Home
Mueller, 2007 [47]	Pre-post study	50	302^t,u^	HH: 95 iHH: 102 QA: 43 Scotoma: 6 Diffuse effect: 48 Tunnel vision: 8	Unclear	3(4)	Stimulation of impaired visual field^q^	60	144	Daily, 6 days a week	6 months	Home
Reinhard, 2005 [48]	Pre-post study	60	15	HH: 9 iHH: 2 QA: 2 iQA: 1 Paracentral scotoma: 1	Left: 7 Right: 8	At least 12	Stimulation of impaired visual field^q^	60	180	Daily, 6 days a week	6 months	Home
Sabel, 2004 [49]	Pre-post study	75	16	HH: 9 iHH: 7	Left: 8 Right: 8	41, 15–127	Stimulation of impaired visual field^q^	30	365	Twice a day, daily	6 months	Home
Mueller, 2003 [50]	Pre-post study, retrospective	50	69^v^	HH: 25 iHH: 22 QA: 10 Scotoma: 3 Diffuse effect: 8 Tunnel vision: 1	Unclear	Unclear	Stimulation of impaired visual field^q^	30	/	Twice a day	6 months	Home
Diabert-Nido, 2021 [51]	Case report	90	1	HH:1	Left:1	156	Audiovisual stimulation of impaired visual field	15	19	Every 2 days	7 weeks	Home
Grasso, 2016 [52]	Pre-post study, with FU	60	10	HH: 10	Left: 4 Right: 6	6, 3–12	Audiovisual stimulation of impaired visual field	240	/	Daily	10 days	Supervised
Keller, 2010^r^ [42]	Controlled trial	80	10	HH: 7 QA: 3	Left: 6 Right: 4	2, 1–6	Audiovisual stimulation of impaired visual field	30	20	/	3 weeks	/
Passamonti, 2009 [53]	Case-controlled	70	12	HH: 12	Left: 6 Right: 6	58, 0.5–360	Audiovisual stimulation of impaired visual field	240	/	Daily	2 weeks	Lab
Plow, 2012b^w^ [54]	Pre-post study, multiple measurements	70	12	HH: 7 QA: 5	Left: 4 Right: 8	39(16)	Stimulation of impaired visual field combined with NIBS^q^	30	/	Twice a day, 3 days a week	3 months	Lab
Plow, 2011 [55]	Case report, 2 cases	60	2	HH: 2	Right: 2	Unclear	Stimulation of impaired visual field combined with NIBS^q^	30	72	3 days a week	3 months	Lab
Räty, 2021 [56]	RCT, double-blind	55	24	HH: 24	Unclear	More than 6 months	NIBS	20–40	10	5 days a week	2 weeks	Darkened room eyes closed
			18	HH: 18	Unclear	More than 6 months	NIBS	20–40	10	5 days a week	2 weeks	Darkened room eyes closed
Rowe, 2017^ m^ [22]	RCT	80	26	HH: 15 iHH; 11	Left: 17 Right: 9	75.5(45), 0–173	Application of external aids	120	42	Twice a day, daily	6 weeks minimum	/
Bowers, 2008 [57]	Pre-post study with FU	75	42^d^	HH: 42	Left: 21 Right: 21	G1: 19, 8–48 G2: 31, 7–60	Application of external aids	As much as possible	As much as possible	As much as possible	At least 4 weeks wearing both prism segments	Home
Selivanova, 2019 [58]	Prospective cohort	95	5^x^	HH: 5	Unclear	Unclear	Multidisciplinary visual rehabilitation	/	/	/	Up to 11 h	Home or clinic
Smaakjaer, 2018 [59]	Pre-post study	75	18^y^	HH: 18^z^	Unclear	9 median, 5–31	Multidisciplinary visual rehabilitation	20 (home) 45 (clinic)	/	Twice a week or once a week	6 weeks or 10–12 weeks	Home or clinic

*RCT* Randomized Controlled Trial, *HH* homonymous hemianopia, *QA* homonymous quadrantanopia, *iHH* incomplete homonymous hemianopia, *iQA* incomplete homonymous quadrantanopia, *FU* follow-up, *UK* United Kingdom, *USA* United States of America, *NIBS* non-invasive brain stimulation, *HVFD* homonymous visual field defect, *G1* group 1, *G2* group 2

^a^See also Appendix A

^b^This reference employed a crossover design. Participants in this study were divided into three groups, all receiving systematic scanning training. Thirty-three participants (‘group 1’) received visual scanning training first and reading training second. Thirty-one participants (‘group 2’) received reading training first and visual scanning training second. Thirty-three participants received a period of detailed advise, followed by visual scanning training and reading training. Due to the aim of the current review, the 97 participants are presented together

^c^Identical sample reported in the same record (Hazelton et al., 2020). One of the 11 participants has visual neglect. Amount of participants enrolled in the intervention differed: 10 participants followed the RI and 11, 9 and 8 participants followed the other interventions

^d^Two subsamples were created in these references. Due to the purpose of this review, the results are presented together, with the exception of time since lesion since this information was only given for the two subgroups separately

^e^Identical sample reported in the same reference (Schuett & Zihl, 2013)

^f^Identical sample reported in the same reference (Schuett et al., 2012)

^g^Participants in this study were divided into two groups. Eleven participants received two blocks of reading intervention (‘group 1’), and 8 participants (‘group 2’) received one block of sham intervention and one block of reading intervention. Due to the aim of the current review, the 19 participants are presented together

^h^The total studied sample in this study consisted of 14 participants. Due to the purpose of the current review, only the 2 participants with a HVFD are presented

^i^Within this intervention, a cross-over design of two types of feedback (internal visual feedback and internal visual feedback concurrent with laboratory-based external auditory oculomotor feedback) was employed

^j^In a similar record by Zihl (1984), the same intervention studied in a part (*n* = 30) of the reported sample in this reference is given. Therefore, only this reference by Zihl (1988) is included in this table

^k^Of these 21 individuals, 11 underwent the experimental training and the other 10 control training. It cannot be extracted which VFD characteristics belong to which individual or group, so information on all 21 individuals is presented in this table

^l^The total studied sample in this study consisted of 9 participants. Due to the purpose of the current review, only the 8 participants with a HVFD are presented

^m^Same reference, two different subsamples and different interventions (Rowe et al., 2017)

^n^Identical intervention studied in two partly different samples

^o^Same reference, two different subsamples and different interventions (Mödden et al., 2012)

^p^Participants in this study were divided into two groups both receiving adjusted saccadic behaviour training. Twenty-one participants received exploration training (‘group A’), and 21 participants received first-attention training and afterward exploration training (‘group B’). Due to the aim of the current review, the 42 participants are presented together

^q^This intervention was carried out within the NovaVision incorporation

^r^Same reference, two different subsamples and different interventions (Keller & Lefin-Rank, 2010)

^s^This reference employed a crossover design. Participants in this study were divided into two groups both receiving stimulation of the impaired visual field. Eight participants received extrastriate vision restitution training (‘group 1’), and 10 participants received conventional vision restitution training (‘group 2’). Due to the aim of the current review, the 18 participants are presented together

^t^In a subsample of the 85 included participants, an additional 3 months of the intervention was given. The pre and post-measurements of this group were before and after these 3 months

^u^Of the 302 participants, 44 presented with a heteronymous visual field defect, strabismus or difference in visual acuity and were therefore trained monocularly. It cannot be extracted from the reference what the size of the visual field defects are in the sample (e.g. HH or QA) of 258 participants trained binocularly. Therefore, the 302 participants are all presented in the table to provide the reader with some information on the size of the visual field defects of the sample

^v^Of the 69 participants, 17 presented with a heteronymous visual field defect. It cannot be extracted from the reference what the size of the visual field defect is of the 52 people with a HFVD in the sample (e.g. HH or QA). Therefore, the 69 participants are all presented in the table to provide some information on the size of the visual field defects of the sample

^w^The included record of Plow et al. (2012b) describes the exact same sample and intervention as this reference and is therefore not listed separately in this table

^x^The total studied sample in this reference consisted of 109 participants. Due to the purpose of the current review, only the 5 participants with a HFVD are presented

^y^The total sample size in this reference is 24, but in 18 participants, information on reading performance could be obtained. Due to the purpose of the current review, only these 18 participants with a HFVD are presented

^z^In record only referred to as ‘hemianopia’

Sample sizes range between 1 and 302 (240 of these 302 participants had a HVFD). The 59 records together include 1720 participants. Of these, 1073 are individuals described as having homonymous hemianopia, 215 incomplete homonymous hemianopia, 203 quadrantanopia, 4 incomplete quadrantanopia and 140 other visual field defects (Table 2). Macular sparing or splitting was not considered because it was not mentioned in most of the studies, although it is of essential significance in iwHs (Horton et al., 2021). A left-sided visual field defect is described in 594 individuals, a right-sided visual field defect in 552 individuals. For 534 participants, the side of the visual field defect is unclear from the paper contents. The time since lesion ranges between 0 and 842 months (i.e. 70 years and 2 months).

### Intervention Type

The included records describe 70 interventions (Table 2). Included in this number are unique interventions or similar interventions studied in unique samples. Sham or control interventions were not included. Of these 70 interventions, 21 can be identified as RI and 49 as OI. Based on the stated mechanisms of the interventions, we distinguished the following categories: (A) adjusted saccadic behaviour (36 interventions in total; 15 RI, 21 OI); (B) stimulation of impaired visual field (16 OI); (C) stimulation of impaired visual field combined with Non-Invasive Brain Stimulation (NIBS; two OI); (D) audiovisual stimulation of the impaired visual field (four OI); (E) multidisciplinary visual rehabilitation (one including reading exercises, two OI); (F) utilisation of the intact visual field (two RI; with utilisation of the intact visual field, compensatory strategies not aimed at oculomotor adaptations are meant, in the included cases specifically, text rotation training was employed); (G) application of external aids (two OI), H) multiple oral re-reading combined with NIBS (two RI), (I) NIBS (two OI) and (J) audiovisual word recognition training (one RI).

### Country of Data Collection

In Table 3, an overview is given of the countries where the interventions took place. Most HVFD intervention research including a reading outcome measure has been conducted in Germany, followed by The Netherlands, the United Kingdom and the United States of America.

**Table 3:** References grouped by intervention type and country of data collection

Country	No.	Intervention type	References (Appendix A)^a^
Germany (total: 25)	1	Adjusted saccadic behaviour	**1**,** 5**^**b**^, **7**^**b**^, **8**, **9**, **12**, **13**, **14**, 1^b^, 5^b^, 6^b^, 29^b^, 31, 33, 34
	11	Stimulation of impaired visual field	29^b^, 40, 42^b^, 43, 44, 45, 46, 47^b^, 48, 49, 50
	1	Audiovisual stimulation of impaired visual field	42^b^
	1	NIBS	56^b^
	1	Utilisation of intact visual field	**15**
The Netherlands (total: 7)	5	Stimulation of impaired visual field	36, 37, 38, 39, 41
	2	Adjusted saccadic behaviour	23, 25
UK (total: 7)	7	Adjusted saccadic behaviour	**2**^**b**^, **4**, **6**, **10**, 2^b^, 22^b^, 30
	1	Application of external aids	22^b^
	1	Audiovisual word recognition training	**20**
USA (total: 7)	2	Adjusted saccadic behaviour	**11**, 26
	2	Stimulation of impaired visual field with NIBS	54, 55
	1	Application of external aids	57
	1	Multidisciplinary visual rehabilitation	58
	1	Multiple oral re-reading combined with NIBS	**18**
Australia (total: 3)	3	Adjusted saccadic behaviour	21, 28, 34
Italy (total: 4)	2	Audiovisual stimulation of impaired visual field	52, 53
	1	Adjusted saccadic behaviour	32
	1	NIBS	56^b^
Austria (total: 3)	4	Adjusted saccadic behaviour	**5**^**b**^, **7**^**b**^, 5^b^, 7^b^
	1	Stimulation of impaired visual field	47^b^
France (total: 2)	2	Adjusted saccadic behaviour	24, 27
Switzerland (total: 2)	1	Stimulation of impaired visual field	47^b^
	1	Utilisation of intact visual field	**16**
Canada (total: 1)	1	Audiovisual stimulation of impaired visual field	51
Denmark (total: 1)	1	Multidisciplinary visual rehabilitation	59
Finland (total: 1)	1	NIBS	56^b^
Malaysia (total: 1)	1	Multiple oral re-reading combined with NIBS	**17**
Mexico (total: 1)	1	Adjusted saccadic behaviour	**3**
Spain (total: 1)	1	Multidisciplinary visual rehabilitation	**19**

*UK* United Kingdom, *USA* United States of America

^a^Bolded references reflect records including reading interventions

^b^This record included multiple intervention types and/or is data was collected in multiple countries and is therefore included multiple times in the tables

### Intervention Characteristics

#### Session Duration

Session duration was reported in 63 out of the 70 interventions and ranges from 10 to 240 min, next to one record reporting ‘as much as possible’ (see Table 2). A categorisation with references grouped by session duration can be found in Table 4. Most frequent in RIs are sessions lasting between 31 and 60 min, whereas in OIs session, a duration of 10–30 min is most reported.

**Table 4:** References grouped by intervention type and session duration (*N* = 63)

Session duration	No.	Intervention type	References (Appendix A)
Reading interventions			
10–30 min	3	Adjusted saccadic behaviour	2^a^, 6, 10
	1	Audiovisual word recognition training	20
	1	Utilisation of intact visual field	15
31–60 min	10	Adjusted saccadic behaviour	1^a^, 4, 5^a^, 7^a^, 8, 9^a^, 9^a^, 11, 13, 14
	2	Multiple oral re-reading with NIBS	17, 18
	1	Multidisciplinary visual rehabilitation	19
120–180 min	1	Adjusted saccadic behaviour	3
Other interventions			
10–30 min	8	Stimulation of impaired visual field	29^a^, 37, 40, 42^a^, 44, 45, 49, 50
	7	Adjusted saccadic behaviour	2^a^, 22^a^, 24, 27, 29^a^, 31, 33
	2	Audiovisual stimulation of impaired visual field	42^a^, 51
	2	NIBS	56^a,b^, 56^a,b^
	2	Stimulation of impaired visual field with NIBS	54, 55
	1	Multidisciplinary visual rehabilitation	59
31–90 min	9	Adjusted saccadic behaviour	1^a^, 2^a^, 2^a^, 5^a^, 7^a^, 23, 25, 28, 30
	8	Stimulation of impaired visual field	36, 38, 39, 41, 43, 46, 47, 48
120–180 min	1	Application of external aids	22^a^
240 min	2	Audiovisual stimulation of impaired visual field	52, 53
	1	Adjusted saccadic behaviour	32
As much as possible	1	Application of external aids	57

^a^This record includes multiple interventions for which session duration is mentioned and is therefore included multiple times

^b^20–40 min

#### Number of Sessions

The number of sessions of an intervention could be derived from 56 out of the 68 interventions (Table 2). The references grouped by intervention type and number of sessions can be found in Table 5. The lowest amount of reported sessions is one, and the highest amount is 300. For RI, most interventions include 5–12 sessions, whereas OI include mostly 12–24 sessions. One record reports a different amount of sessions for individuals with left-sided and right-sided HVFDs.

**Table 5:** References grouped by intervention type and number of sessions (*N* = 56)

Number of sessions	No.	Intervention type	References (Appendix A)
Reading interventions			
5–12 sessions	7	Adjusted saccadic behaviour	1^a^, 2^a^, 5^a^, 7^a^, 9^a^, 9^a^, 12^b^
	2	Stimulation of impaired visual field with NIBS	17, 18
14–20 sessions	3	Adjusted saccadic behaviour	10, 11, 13
21–42 sessions	2	Adjusted saccadic behaviour	8, 12^b^
	1	Audiovisual word recognition training	20
	1	Utilisation of the intact visual field	15
120–168 sessions	1	Multidisciplinary visual rehabilitation	19
Other interventions			
1–12 sessions	8	Adjusted saccadic behaviour	1^a^, 2^a^, 2^a^, 5, 7^a^, 24, 27, 33
	2	NIBS	56^a^, 56^a^
14–24 sessions	9	Adjusted saccadic behaviour	2^a^, 21, 23, 25, 26, 28, 29^a^, 30, 32
	2	Audiovisual stimulation of impaired visual field	42^a^, 51
	2	Stimulation of impaired visual field	29^a^, 42^a^
40–72 sessions	5	Stimulation of impaired visual field	38, 39, 41, 43, 46
	2	Adjusted saccadic behaviour	22^a^, 31
	1	Application of external aids	22^a^
	1	Stimulation of impaired visual field with NIBS	55
144–365 sessions	5	Stimulation of impaired visual field	40, 45, 47, 48, 49
Multiple sessions a day / as much as possible	2	Stimulation of impaired visual field	36, 37
	1	Application of external aids	57

^a^This record includes multiple interventions for which the number of sessions is mentioned and is therefore included multiple times

^b^11 sessions for individuals with left-sided hemianopia, 22 sessions for individuals with right-sided hemianopia (mentioned two times but counted as 1 in total *N*)

#### Intervention Intensity

For 52 interventions, the intervention intensity is reported (Table 2). The references categorised by intervention type and intervention intensity can be found in Table 6. The lowest intervention intensities are one session and a session every 4–5 weeks (both OIs). The highest intervention intensity consists of five 12-min sessions per day (OI). In the majority of interventions, 41 cases, one to three daily intervention sessions are reported. *Daily* meaning either 5, 6 or 7 days a week. Of these 41 interventions, 15 are RI, and 26 are OI. Eleven records report on one to three sessions per week, of which four RI and seven OI. One OI suggests the intervention intensity to be “as much as possible”.

**Table 6:** References grouped by intervention type and intervention intensity (*N* = 50)

Intervention intensity	No.		Intervention type	References (Appendix A)
Reading interventions				
1–3 sessions a day^a^	11		Adjusted saccadic behaviour	1^b^, 2^b^, 4, 6, 7^c^, 9^b^, 9^b^, 10, 12, 13, 14^c^
	1		Audiovisual word recognition training	20
	1		Multidisciplinary visual rehabilitation	19
	1		Multiple oral re-reading combined with NIBS	18
	1		Utilisation of the intact visual field	15
1–3 sessions a week	4		Adjusted saccadic behaviour	3, 5, 11, 14^c^
Other interventions				
As much as possible	1	Application of external aids		57
1–3 sessions a day^a^	13	Stimulation of impaired visual field		36, 37, 38, 40, 41, 43, 44, 45, 46, 47, 48, 49, 50
	6	Adjusted saccadic behaviour		1^b^, 2^b^, 22^b^, 31, 32, 35^d^
	3	Audiovisual stimulation of impaired visual field		51, 52, 53
	2	NIBS		56^e^, 56^e^
	1	Application of external aid		22^b^
1–3 sessions a week	4	Adjusted saccadic behaviour		26, 28, 33^f^, 35^d^
	2	Stimulation of impaired visual field with NIBS		54^ g^, 55
	1	Multidisciplinary visual rehabilitation		59
Every 4–5 weeks	1	Adjusted saccadic behaviour		24
Once	1	Adjusted saccadic behaviour		27

^a^‘A day’ meaning either 5, 6 or 7 days a week

^b^This record includes multiple interventions for which intervention intensity is mentioned and is therefore included multiple times

^c^Usually daily, at least 3 times per week (mentioned two times but counted as one in total *N*)

^d^Daily or 3 times a week (mentioned two times but counted as one in total *N*)

^e^Four to six sessions per week

^f^Twice a week, 2 times a day

^g^Twice a day, 3 days a week

#### Intervention Duration

Intervention duration is reported in 62 interventions (Table 2). A categorisation with references grouped based on the intervention duration can be found in Table 7. Intervention duration ranges between 1 day and 9 months for RI and 1 day and 6 months for OI. Within RIs, interventions lasting 2–4 weeks and interventions lasting 5–12 weeks occur in a similar amount, both 7 times. For OIs, slightly more interventions lasting 5–12 weeks are reported compared to interventions lasting 2–4 weeks (18 and 16 times, respectively).

**Table 7:** References grouped by intervention type and intervention duration (*N* = 62)

Intervention duration	No.	Intervention type	References (Appendix A)
Reading interventions			
1 day to 1–2 weeks	2	Adjusted saccadic behaviour	6, 12
	1	Utilisation on the intact visual field	17
	1	Multiple oral re-reading with NIBS	18
2–4 weeks	6	Adjusted saccadic behaviour	2^a^, 5^a^, 7^a^, 9^b^, 9^b^, 13^c^
	1	Utilisation on the intact visual field	15
5–12 weeks	4	Adjusted saccadic behaviour	4, 10, 11, 13^c^
	1	Audiovisual word recognition training	20
	1	Multiple oral re-reading with NIBS	17
	1	Multidisciplinary visual rehabilitation	19
9 months	1	Adjusted saccadic behaviour	3
Other interventions			
1–10 days	1	Adjusted saccadic behaviour	27
	1	Audiovisual stimulation of impaired visual field	52
	1	Multidisciplinary visual rehabilitation	58
2–4 weeks	7	Adjusted saccadic behaviour	2^a^, 5^a^, 7^a^, 29^a^, 30, 32, 33
	4	Stimulation of impaired visual field	29^a^, 42^a^, 43, 46
	2	Audiovisual stimulation of impaired visual field	42^a^, 53
	2	NIBS	56^a^, 56^a^
	1	Application of external aids	57
5–12 weeks	7	Adjusted saccadic behaviour	2^a^, 21, 22^a^, 23, 25, 28, 31
	6	Stimulation of impaired visual field	36, 37, 39, 40^d^, 41, 44
	2	Stimulation of impaired visual field with NIBS	54, 55
	1	Application of external aids	22^a^
	1	Audiovisual stimulation of impaired visual field	51
	1	Multidisciplinary visual rehabilitation	59
13 weeks–6 months	7	Stimulation of impaired visual field	38, 40^d^, 45, 47, 48, 49, 50
	1	Adjusted saccadic behaviour	24

^a^This record includes multiple interventions for which intervention duration is mentioned and is therefore included multiple times

^b^This record includes two interventions and is therefore included twice

^c^4–6 weeks (mentioned two times but counted as one in total *N*)

^d^3 or 6 months (mentioned two times but counted as one in total *N*)

#### Intervention Circumstance

In 55 of the 70 interventions, information is given about the circumstances under which the intervention was given (Table 2). In Table 8, the references are grouped by intervention type and circumstance. Twenty-six interventions are home-based or self-administered of which seven RI and 19 OI. Sixteen interventions took place in a professional environment such as the clinic, hospital or lab. Of these 16, two are RI and 14 are OI. For 14 interventions, it can be derived that intervention sessions were supervised (no statement about intervention location). Six of the supervised interventions were RI, and eight of the supervised interventions were OI.

**Table 8:** References grouped by intervention circumstance and intervention type (*N* = 53)

Circumstance	No.	Intervention type	References (Appendix A)
Reading interventions			
Home-based/self-administered	4	Adjusted saccadic behaviour	2^a^, 4, 6,10
	1	Audiovisual word recognition training	20
	1	Multidisciplinary visual rehabilitation	19^b^
	1	Utilisation of the intact visual field	15
Clinic/hospital/lab	1	Adjusted saccadic behaviour	11
	1	Utilisation of the intact visual field	16
Supervised	6	Adjusted saccadic behaviour	1^a^, 5, 7, 9^a^, 9^a^, 13
Other interventions			
Home-based/self-administered	10	Stimulation of impaired visual field	36, 37, 38, 40, 43, 46, 47, 48, 49, 50
	5	Adjusted saccadic behaviour	2^a^, 2^a^, 2^a^, 30, 31
	2	Multidisciplinary visual rehabilitation	58^c^, 59
	1	Application of external aids	57
	1	Audiovisual stimulation of impaired visual field	51
Clinic/hospital/lab	7	Adjusted saccadic behaviour	23, 24, 25, 26, 27, 28, 33
	2	Stimulation of impaired visual field with NIBS	54, 55
	2	NIBS	56^a^, 56^a^
	1	Audiovisual stimulation of impaired visual field	53
	1	Multidisciplinary visual rehabilitation	58^c^
	1	Stimulation of impaired visual field	45
Supervised	4	Adjusted saccadic behaviour	1^a^, 29, 32, 34
	3	Stimulation of impaired visual field	29, 39, 41
	1	Audiovisual stimulation of impaired visual field	52

^a^This record includes multiple interventions for which intervention circumstance is mentioned and is therefore included multiple times

^b^Four sessions in-office, the rest home-based

^c^Home or clinic (mentioned two times but counted as one in total *N*)

### Reading Measures

#### Task Performance Measures

From the 59 included records, 49 included task-performance measures for measuring reading (Table 9; 20 RI, 29 OI). Most often an unpublished paragraph reading task is used. These texts originate for example from storybooks or newspapers (e.g. references 5, 20 or 43). Seventeen times this paragraph task was catagorised as being standardised. In 13 cases, this was because the task was described as such by the authors of that record (references 1, 5, 6, 7, 9, 10, 11, 14, 27, 35, 39, 41, 42). In four cases, this choice was made because the same reading task was read by a small sample of healthy control participants (references 12, 13, 20 and 30). Additionally, in two of these references there was also made use of parallel versions of the paragraph (references 13 and 20). Following standardised paragraphs in reported frequency are unstandardised (and unpublished) paragraph tasks. Subsequently, published reading tests are mentioned in the included literature, most often the International Reading Speed Texts (IReST; Trauzettel-Klosinski & Dietz, 2012) followed by the Radner reading chart (Radner et al., 2002), Minnesota Low-Vision Reading Test (MNREAD; Legge et al., 1989), Pepper Visual Skills for Reading Test (VSRT; Stelmack et al., 1987), Western Aphasia Battery (Kertesz, 2007), a subtask of the Wechsler Memory Test (Wechsler, 2004) and the Stroop task (Periáñez et al., 2021; Stroop, 1935).

**Table 9:** Measurement of reading, task performance measures and outcome measures

	No. of references	References (Appendix A)
Total	49	**1**, **2**, **3**, **4**, **5**, **6**, **7**, **8**, **9**, **10**, **11**, **12**, **13**, **14**, **15**, **16**, **17**, **18**, **19**, **20**, 21, 22, 24, 25, 26, 27, 28, 29, 30, 31, 32, 34, 35, 36, 37, 39, 40, 41, 42, 44, 46, 48, 51, 52, 53, 54, 55, 56, 59
Standardised paragraph * WPM* * Number of errors* * Reading time* * Eye tracking* * Accuracy (correct responses)* * Correct answers*	17 10 8 7 6 1 1	**1**, **5**, **6**, **7**, **9**, **10**, **11**^**a**^, **12**, **13**, **14**, **20**, 27, 30, 35, 39, 41, 42 **1**, **5**, **7**, **9**, **10**, **20**, 27^a^, 30, 39, 41^a^ **1**, **5**, **7**, **9**, **14**, 13, 30, 35 **6**^**b**^, **12**, **13**, **14**, 30, 35, 42 **9**^**c**^, 1**0**, **11**, **12**, 39, 41 **20** **11**^**f**^
Paragraph * WPM* * Syllables per second* * Reading speed* * Correct answers* * % increased speed* * Number of errors*	10 7 2 2 2 1 1	**4**, **18**, 24, 25^d^, 36, 37, 48, 52, 53, 59 **4**, 24, 25^d^, 36^e^, 37^e^, 48, 59 52, 53 **18** 25^d^ 36^ g^ **4**
IReST * WPM* * Number of errors* * % change in wpm*	6 6 1 1	**2**, **15**, **16**, **19**, 31, 56 **2**^**h**^, **15**, **16**, **19**, 31, 56 16 16
Radner reading chart * LogRAD* * WPM* * Number of errors* * Correct answers*	4 3 2 2 1	22, 25^d^, 40, 44 25^d^, 40, 44^i^ 25^d^, 40 22, 44 25^d^
Single word reading * Reading time* * Eye tracking* * Correct responses* * Mean reading speed*	4 2 2 2 1	**10**, **12**, **20**, 32 **10**, **12** **10**, **12** **10**, 32 **20**
Unclear * Reading behaviour observations*^*k,l*^ * WPM* * Number of errors*	4 2 1 1	**3**^**j**^, 8^**j**^, 26, 34 **3**^**j,k**^, 26^ l^ **8**^**j**^ **8**^**j**^
MNREAD * WPM*	3 3	51, 54, 55 51, 54^ m^, 55
VSRT * Reading speed* * Unclear*	2 1 1	21, 28 21 28
Stroop task * Time to perform trial*	1 1	46 46
Text from the Wechsler Memory Test * WPM* * Number of errors*	1 1 1	29 29 29
Letter reading * Reading time* * Correct responses*	1 1 1	**20** **20** **20**
Western Aphasia Battery * Reading component total score* * Reading score*	1 1 1	**17** **17** **17**

Bolded references indicate a reference including a RI

*WPM* words per minute, *IReST* International Reading Speed Texts, *WMT* Wexler Memory Test, *VSRT* Pepper Visual Skills Reading Test

^a^Silently read

^b^Reading time presented as the mean of three paragraphs read by the participant.

^c^Data available for a subsample of 14 participants

^d^References by de Haan et al. (2015a, b; reference 25 Appendix A) and de Haan et al. (2016; reference 23 Appendix A) from the reference list employ the same study protocol and sample, therefore, only reference 25 is included in this table

^e^Words per minute calculated based on the first and last saccade while reading the paragraph

^f^Yes/no comprehension questions

^g^Based on the first and last saccade while reading the paragraph

^h^‘Reading speed’ is mentioned as the outcome measure in this reference; however, because the well-described IReST test is employed, we make the assumption that the authors analysed WPM

^i^Presumable LogRad score referred to in this reference as the ‘Radner reading score’

^j^This reference has the design of a case study. It appears that the measures for reading performance are incorporated in the described intervention

^k^Reported reading behaviour observations are reading mistakes, rhythm and intonation

^l^Reported reading behaviour observations are rereading lines, skipping words and using finger to keep in place

^m^Words per minute presented for three different print sizes

#### Task Performance Outcome Measures

In total, 89 times a task performance-based reading outcome measure is reported (Table 9). Most reading outcome measures focus on the time it takes a participant to read (in 49 cases), e.g. reading time and reading speed (measured in words per minute or other units). In other cases, outcome measures are the number of errors/correct responses, eye tracking measures (e.g. amount of forward or backward saccades during reading), the Radner reading acuity score (LogRAD; Radner et al., 2002) and correct answers about the content of the text. In two cases, observations about the reading behaviour of the participant are described, e.g. ‘skipping lines’ or ‘better intonation’. In one record, the reading component total score and reading score of the Western Aphasia Battery were reported.

#### Self-Report Measures

Of the 59 records, 28 report one or more self-report measures of reading (10 RI, 18 OI; Table 10). Most often, authors develop a number of questions or modify an existing questionnaire to fit their (reading-related) research question(s). Following are records describing reading difficulties spontaneously mentioned by participants, e.g. by using a Goal Attainment Scale (GAS) or while conducting interviews. Reading-related items or scales of the following questionnaires are reported: the reading scale of the Veterans Affair Low-Vision Visual Functioning Questionnaire (VA LV VFQ-48; Stelmack et al., 2004), the reading scale of the Impact of Visual Impairment Questionnaire (IVI; Hassell et al., 2000; Lamoureux et al., 2006), a questionnaire by Kerkhoff and colleagues (1990; without title) which provides information about reading and visual exploration, the reading scale of the Visual Impairments Questionnaire (VIQ; Kerkhoff et al., 1994) and the reading item from the 25-item version of the National Eye Institute Visual Function Questionnaire (NEI-VFQ-25; Mangione et al., 2001). Furthermore, 10 records report the use of questionnaires which contain reading items or reading scales but do not specifically report about these reading items or scales (Table 10).

**Table 10:** Measurement of reading, self-report measures and outcome measures

	No. of references	References (Appendix A)
Total	28	**1**, **2**, **3**, **4**, **5**, **6**, **7**, **9**, **11**, **15**, 22, 26, 28, 30, 32, 33, 35, 38, 43, 46, 47, 48, 49, 50, 51, 57, 58, 60
Self-developed or modified question(s)	11	**1**, **5**, **6**, **11**, **15**, 30^b^, 32, 33, 46, 48, 49
Spontaneous reports or (semi-structured) interviews	10	**2, 3,** 26, 35, 38, 43, 47, 49, 50, 57
VA LV VFQ-48 * Reading scale*	4 4	22, 28, 51, 60 22, 28, 51, 60
IVI * Reading scale*	2 2	**15**, 58 **15**, 58
Questionnaire by Kerkhoff et al. (1990) * Reading and visual exploration*	2 2	**7**, **9** **7**, **9**
VIQ * Reading scale*	1 1	**4**^**b**^ **4**^**b**^
NEI-VFQ-25 * Reading item*	1 1	**4** **4**

Bolded references indicate a reference including a RI

*NEI-VFQ-25* 25-item National Eye Institute Visual Function Questionnaire, *VA LV VFQ-48* 48-item Veterans Affairs Low Visual Functioning Questionnaire, *VIQ* Visual Impairments Questionnaire.

- References **2**, **19**, 21, 24, 25, 28, 44, 45, 58 and 60 are included in this review due to the inclusion of a self-report reading measure, but this measure is not specifically mentioned in this table. These references have stated to administer the VA LV VFQ-48 (**2**) or IVI (60), both of which contain a reading scale. These references have not specifically reported that scale, but we can assume that the authors have collected information about reading by administering these questionnaires and are therefore included in the table. References **19**, 21, 24, 25^a^, 28, 44, 45 and 58 report the use of the NEI-VFQ-25. One of the 25 items of the NEI-VFQ-25 concerns reading. Studies that report either the composite score (24, 25^a^), near vision scale (including the reading item; **19**, 21, 28, 44, 45) or visual functioning scale (58) are mentioned in this table on the premise that they have collected information on reading through that item. These references have not reported specifically about that item

^a^References by de Haan et al. (2015a, b; reference 25 Appendix A) and de Haan et al. (2016; reference 23 Appendix A) from the reference list employ the same study protocol and sample; therefore, only reference 25 is included in this table

^b^Possibly the same questionnaires. In reference 4, there is referred to, amongst others, reference 29, but in the latter reference, the term ‘VIQ’ is not used

Out of the 59 records, 23 include the use of both task performance and self-report measures of reading (references 1, 2, 3, 4, 5, 6, 7, 9, 11, 15, 19, 22, 24, 25, 26, 28, 30, 32, 35, 44, 46, 48, 51). Of these 23 records, 11 are RIs, and 12 are OIs.

### Quality Assessment

**Fig. 2:**
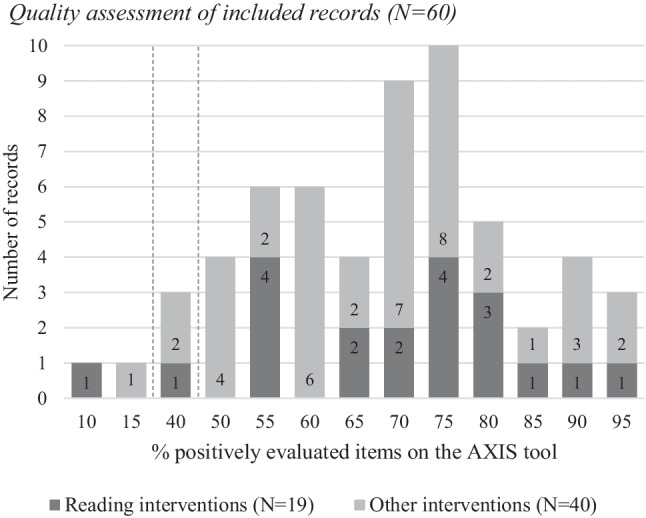
Quality assessment of included records (*N* = 60)

The percentage of positively rated items on the AXIS tool ranges between 10 and 95% (Fig. 2; for a complete overview of the quality assessment, see Table 2 and Appendix B). The mean obtained positively rated items of all studies is 67%. Studies including RIs and studies including OIs do not appear to differ considerably in quality. Keeping the aim of this review in mind, we would like to highlight results on a few specific items of the AXIS tool. Item 8 evaluates the appropriateness of the used measurement tools (Downes et al., 2016). On this item, for 18 (90%) out of the 20 records investigating (also) a RI, a positive evaluation (‘1’) was scored. In OIs item, 8 was positively evaluated in 31 (78%) out of the 40 records. Thus, most RI and OI used an appropriate measurement tool for reading. However, the reliability of the used measurement tools was in most cases rated negatively, as is reflected by the scores on item 9 (Downes et al., 2016). This item received a positive evaluation in 9 (45%) out of the 20 RI records. Similarly, for OIs item, 9 was positively evaluated in 16 (40%) out of the 40 records. The interrater reliability is 79%.

## Discussion

### Reviewing Interventions

The current systematic review is the first to provide an overview of all HVFD intervention research in which reading was measured, as well as to provide insights into the different reading outcome measures these studies have adopted. We included 60 records in this review. The majority of the included records employed a pre-post study design, followed by RCTs, case reports and case control studies. In total, 70 interventions could be identified that were either unique from each other or similar but studied in unique samples. Twenty-one interventions were primarily aimed at alleviating reading difficulties in iwHs. Forty-nine interventions were not primarily aimed at improving reading, but nevertheless, the effect on reading was included in the secondary outcome measures. A majority of records reported on the intervention characteristics of our interest, making it possible to provide an overview that can possibly guide clinicians, researchers and interested iwHs to studies of interest with regard to these factors. Overall, adjusted saccadic behaviour interventions were described most in the literature. Additionally, one-third of the HVFD intervention research appears to have (also) taken place in Germany. We included the country in which a study took place as a contextual factor of an intervention. It should, however, be noted that next to being dependent on geographical components such as public health leaders and funding opportunities, the success rate of implementing an intervention into clinical practice is also influenced by other components like organisational culture and stakeholders (Brownson et al., 2009).

**Fig. 3:**
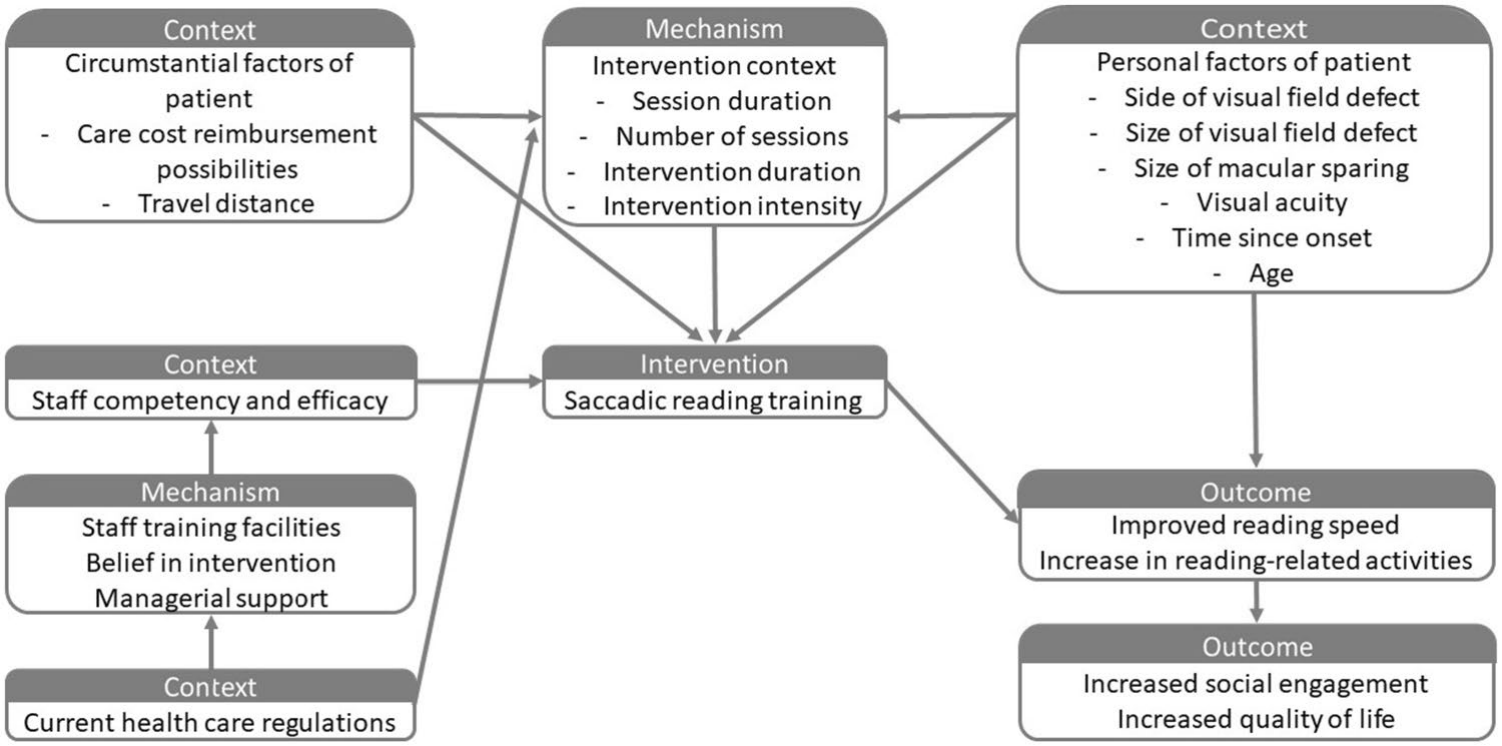
Fictional logic model of programme theory for a saccadic reading training

Intervention characteristics help to understand the context in which an intervention may be effective (O’Cathain et al., 2019). In their guidance papers for developing and evaluating complex interventions, the Medical Research Council have been giving increasing attention to the need for understanding how and under what condition interventions can generate change (e.g. Greenhalgh & Papoutsi, 2018; Moore et al., 2015; Skivington et al., 2021). This understanding can also be referred to as the *programme theory* of an intervention (O’Cathain et al., 2019; Rogers, 2008). An example of developing a programme theory has been provided by Handley and colleagues (2017). By conducting interviews with stakeholders and synthesising evidence from the literature, the authors developed a programme theory about outcomes of hospital care for individuals living with dementia. The authors took into account contextual factors within patients, staff and the hospital environment. By doing so, they showed e.g. the necessity of support for staff members and availability of resources for actually improving patient outcomes. Previous reviews have called for more high-quality studies on the topic of effectiveness of HVFD interventions (Bouwmeester et al., 2007; Hanna et al., 2017; Pollock et al., 2019), we would like to add to this call attention for understanding the programme theory of HVFD interventions in order to develop a full picture on the possibilities of an intervention. A fictional and visual example of a programme theory can be seen in Fig. 3. Note that there are multiple ways a programme theory could be developed and visualised (see examples in Handley et al., 2017; O’Cathain et al., 2019; Stephens et al., 2018).

Though identifying the programme theory of included interventions was not one of the main goals of the current review, we have provided an overview of possible relevant conditions of published interventions. Evidently, intervention characteristics can be very relevant to the iwH or clinician on for example a geographical or social level (O’Cathain et al., 2019). To illustrate, iwHs can have difficulties with independent mobility (de Haan et al., 2015a, b), which can alter the practical rehabilitation possibilities for these individuals. Conveniently, included in our overview is the existing literature of HVFD interventions which are self-administered and thus not requiring the individual to travel for treatment. A note of caution is due here since readers have to take into account that this review does not give information about the effectiveness of reported interventions. As mentioned, there are multiple recent systematic reviews and meta-analyses on the effectiveness of (reading) interventions for iwHs available (e.g. Hanna et al., 2017; Liu et al., 2019; Maeyama et al., 2023; Pollock et al., 2019). Based on the limited evidence, reviews have pointed to the direction of compensatory interventions (e.g. saccadic adaptation or utilisation of the seeing hemifield) as the only empirically supported interventions for iwH at present (Bouwmeester et al., 2007; Hanna et al., 2017; Pollock et al., 2019). Indeed, a recent systematic review and meta-analysis on HVFD interventions for specifically reading has shown only positive effects of compensatory reading interventions, compared to visual exploration training and visual restoration therapy (Maeyama et al., 2023). For information about the effectiveness of the interventions, we refer to systematic reviews and meta-analysis as well as to the original studies and would like to advise the reader to pay critical attention to the employed study design and representativeness of the study sample of that study to the iwH in question.

### Measurement of Reading

The current review provides an overview of all outcome measures related to reading in HVFD intervention research. The majority of task-performance measures are based on paragraph reading tasks. This seems appropriate, as longer text paragraphs may resemble real-life reading situations more than shorter sentences or words (Brussee et al., 2014, 2021), as well as being more suitable for repeated measurements (Altpeter et al., 2015) and minimising undesired influence of pronunciation errors and correction of reading errors more so than word and short sentence tasks (Rubin, 2013). It has been suggested that paragraphs may be most applicable for individuals with reading speeds above 80 words per minute (Brussee et al., 2014). This however seems to pose no considerable limitation for research with iwHs, judging the mean words per minute of the (sub)samples of the included records (82–137 words per minute; Aimola et al., 2014; Jacquin-Courtois et al., 2013; Kuester-Gruber et al., 2020; Lane et al., 2010; Levy-Bencheton et al., 2016; Mena-Garcia et al., 2021; Räty et al., 2021; Reinhard et al., 2005; Schuett et al., 2008, 2012; Schuett & Zihl, 2013; Smaakjær et al., 2018; Spitzyna et al., 2007; Zihl et al., 2021). Two records should be mentioned as exceptions here, as Bergsma and van der Wildt (2010) note of a mean of 235 words per minute in the sample, and Woodhead and colleagues (2013) note a mean of 63 words per minute. This latter study was however performed with iwHs, and pure alexia in which a lower reading speed can be expected in comparison to iwHs without pure alexia (Pflugshaupt et al., 2009).

Additionally, reading difficulties of iwHs do not exclusively exist on the word-level but certainly on the text-level as well; for example, difficulties with finding the beginning of the next line (Trauzettel-Klosinski & Brendler, 1998), favouring paragraph reading tasks as well. Other critical aspects to consider regarding paragraph reading tasks are that due to the semantic context, complex non-visual aspects play a part in the reading performance, for example cognitive reserve (Rubin, 2013). Additionally, the semantic context could contribute to an overestimation of reading acuity (Brussee, et al., 2015). As mentioned earlier, however, these aspects make paragraph reading tasks more resembling daily life tasks and performance.

The current review also shows that most task-performance outcome measures are time-based, and to a lesser extent, based on reading errors or comprehension of the text. This seems appropriate, as Rubin (2013) in his review on the measurement of reading has concluded that reading speed is a key outcome variable that can predict, amongst others, vision-related quality of life. Indeed, both vision-related quality of life and reading speed measures improved after intervention in the included records of Aimola et al. (2014), Crotty et al. (2018), Hazelton et al. (2020), Kuester-Gruber et al. (2020) and Levy-Bencheton et al. (2016). However, in the studies of de Haan et al. (2016) and Mena-Garcia et al. (2021), an improvement on the NEI-VFQ-25 was found but not on reading speed after intervention. Interestingly, Hazelton and colleagues (2020) observed that although both the IReST and VA LV VFQ-48 scores did not improve after intervention in the experimental groups, the participants did indicate that they experienced benefits from the interventions. Though measures of reading speed appear most used and recommended, reading errors and comprehension measures can be of interest as well, depending on the clinical or research question. For example, the type of reading error can provide information about the type of visual field deficit (Zihl, 2011).

#### Suggestions for Future Research

##### Use of Validated Paragraph Reading Tasks

Though the findings above suggest paragraph reading tasks with reading speed outcome measures as favoured measures in HVFD intervention research, no general consensus on which measures to employ exists. Based on the outcomes of this review, we would like to make three suggestions for future studies with regard to measuring reading in iwHs. The first suggestion is making use of published (paragraph) reading tests with available information on the psychometric properties of that test. The current review shows that although some studies make use of trialled and published tests (e.g. IReST, Radner reading chart, MNREAD), the majority of studies make use of self-developed paragraph reading tasks with no or little information about the validity, reliability and responsiveness of these measures (e.g. newspaper texts). This finding is also reflected in our observation that more than half of the included studies scored negatively on AXIS item concerning instrument reliability (i.e. item 9). Using standardised and validated reading tests would increase comparability between HVFD intervention studies. For an overview of the psychometric properties of text reading tasks, see for example Brussee et al. (2015) and Brussee et al. (2014). For information on psychometric properties of reading tests in individuals with macular pathologies, see Brussee et al. (2021) and Kortuem et al. (2021). Based on these studies, the IReST, Radner reading chart or MNREAD are the best-studied and qualified tests at this moment in time.

##### Employ Mixed-Method Measure of Reading

Our second suggestion is to make use of both task-performance and self-report measures for reading and reading-related intervention outcomes. While the majority of records included task-performance measures of reading, less than half of included records used measures of reading based on self-report. The proportion of records combining both types of reading measures is even smaller. This is surprising since studies have shown the value of mixed-methods design for obtaining a more complete understanding of what is studied in health-related topics (Guetterman et al., 2015). Research in other populations familiar with cognitive difficulties has shown that self-report and task-performance measures of cognition did not correspond (e.g. Fuermaier et al., 2015; Koerts et al., 2012; Vlagsma et al., 2017). These outcomes suggest that task performance and self-report measures represent different levels of functioning and therefore both contribute to the assessment of difficulties. It remains unclear whether the same can be said for reading performance. However, included studies in the current review indicate that the same reading construct (i.e. reading *speed*) measured both objectively and subjectively seem to give similar results (Ong et al., 2012; Schuett et al., 2008, 2012). Overall however, within the small proportion of studies employing a mixed-method design, task-performance measures based on reading speed and most self-reported measures have a broader perspective, namely if the iwHs experienced difficulties with reading (e.g. Aimola et al., 2014; Daibert-Nido et al., 2021; Kuester-Gruber et al., 2020; Lane et al., 2010, Zihl et al., 2021).

##### Facilitate Patient-Relevant Outcome Measures

Bouwmeester and colleagues (2007) have pointed out that validated questionnaires are the most reliable way to measure the subjective transfer of HVFD interventions to daily life activities. Indeed, we found that multiple studies have included quality of life or daily life questionnaires to measure the effect of intervention. These questionnaires can be helpful to gain insight in overall functioning or satisfaction of the iwHs but do not provide a comprehensive overview of reading, as reading in these questionnaires is reflected in one or a few items only. An interesting finding in our review is that most studies including self-report measures used self-developed or modified questions about reading or registered participant’s spontaneous comments about reading, e.g. during interviews. While using self-designed questionnaires has the advantage that the questions are closer to the aims of a specific study, it has the disadvantage that it potentially comes at the expense of the validity of the questions. However, currently, neither consensus on relevant reading measures for iwHs as well as no validated HVFD reading questionnaire exists. It is therefore recommended to include types of measures that provide space for personally relevant outcomes, such as interviews or Goal Attainment Scaling (GAS; Grant & Ponsford, 2014). To give an example, Kersting and colleagues (2020) write that social dimensions are often underrepresented in patient-relevant outcome measures. In the context of lacking (validated) subjective reading measures, an interview or GAS has the potential to provide space for aspects of reading with HVFDs.

## Limitations

We recognise that multiple factors influencing reading performance were not included in our overviews. These factors include individual characteristics such as age and level of education, which have been related to reading performance (Brussee et al., 2016). Additionally, we neither included factors potentially related to reading tasks, such as reading distance or whether text had to be read aloud or silently (Brussee et al., 2016; Rubin, 2013). Relevant individual characteristics that were included comprised of the side and size of the HVFD and time since lesion (Trauzettel-Klosinski & Brendler, 1998). However, the absence or the size of macular sparing was not included in our overviews, which has been shown to be an important factor for reading in iwHs (Horton et al., 2021).

The categorisation of intervention types we chose was not based on an existing or accepted classification system. Other researchers might have come up with a different classification system. Different researchers creating different labels, however, is inherent to qualitative data interpretation (Doleman et al., 2021; Owens, 2021). Additional potential biases specifically related to systematic reviews should also be mentioned, such as selective outcome reporting. By registering our review protocol in a database (see Tol et al., 2020) and adhering to the PRISMA-P guidelines (Moher et al., 2009), we aimed to minimise these biases.

Our systematic search consisted of the inclusion of peer-reviewed studies through three databases, backward citation tracking of the included records, trial register searches and a Google Scholar search. The addition of Google Scholar as a database has been suggested in previous studies (Bramer et al., 2017; Haddaway et al., 2015) and also resulted in one additional included record in the current review. We assessed the first 150 of many hits on Google Scholar. One could argue this might have resulted in missing potential inclusions emerging in further hits. However, we thought it justified to set an (arbitrary) limit of 150 as it also has been shown that searching for grey literature sources can also be inefficient and overly repetitive (Giustini, 2019). Although we used grey literature sources such as protocol registers and Google Scholar to find eligible studies, grey literature itself was not considered for inclusion in this review as we only included peer-reviewed intervention studies. Including grey literature, i.e. not formally published literature such as government papers, data from trial protocols or unpublished data, is commonly advised in reviews aiming to inform practice or policy (Giustini, 2019) and can therefore be considered a limitation of the current review, increasing the risk of providing an unbalanced representation of HVFD interventions (Paez, 2017).

## Conclusion

Main takeaway messages for clinicians, researchers and interested iwHs are summarised in Table 11. The current review provides an overview of all HVFD intervention research reporting on reading performance. Even though compensatory methods, e.g. adjusted saccadic behaviour, are the only empirically supported evidence at present, this overview can guide clinicians, researchers and interested iwHs to all published interventions based on contextual factors and reading outcome measures. For future studies, we suggest to take the development of a programme theory into account in HVFD intervention research, use standardised and validated paragraph reading tasks and include account both task-performance and self-report measures of reading.

**Table 11:** Main take away messages for clinicians, researchers and individuals with HVFDs

Clinicians	You can use this review for reference when you are searching for HVFD intervention possibilities. This review can help you find studies with specific contextual factors (e.g. amount of sessions or electronic possibilities) and also help find certain included reading outcome measures that might be relevant for the individual you are treating (e.g. reading speed or reading-related quality of life). Take into account that this review does not give information about the effectiveness of the reported interventions. For information about the effectiveness, we refer to the original studies. Compensatory methods (adjusted saccadic behaviour) are the only empirically supported methods at present. When evaluating an intervention study, pay attention to the studied sample and measurement methods in order to estimate the value of this intervention for the individual you are treating
Researchers	When studying HVFD interventions and their outcome on reading, preferably choose validated and standardised reading tests to increase comparability between intervention studies when measuring objective reading as well as to make sure you measure what you aim to measure. Including a self-report reading outcome measure next to a task-performance reading measure is advised. Since there are no validated reading questionnaires for iwHs and little to no research on patient-relevant outcome measures in this field, we suggest incorporation interviews or a GAS to provide participants the possibility to supply outcomes relevant to them
iwHs	Reading is an everyday skill, and it can be very debilitating to experience reading difficulties due to a HVFD. You can think of what aspects of reading are important for *you* in your everyday life. This review provides an overview of the different ways to measure reading that studies have chosen to measure the effect of an intervention and can therefore inform you on personally interesting studies Discuss the possibilities with your clinician with regard to interventions to alleviate reading difficulties

*HVFD* homonymous visual field defect, *iwHs* individuals with homonymous visual field defects, *GAS* Goal Attainment Scale

## Data Availability

Data are available upon reasonable request from the corresponding author.

## Appendix A References to included records

**Table:**

**1**	**Zihl et al. (**2021**)**
**2**	**Hazelton et al. (**2020**)**
**3**	**Pineda-Ortiz et al. (**2018**)**
**4**	**Aimola et al. (**2014**)**
**5**	**Schuett and Zihl (**2013**)**
**6**	**Ong et al. (**2012**)**
**7**	**Schuett et al. (**2012**)**
**8**	**Kerkhoff and Marquardt (**2009**)**
**9**	**Schuett et al. (**2008**)**
**10**	**Spitzyna et al. (**2007**)**
**11**	**Ciuffreda et al. (**2006**)**
**12**	**Zihl (**1995**)**
**13**	**Kerkhoff et al. (**1992**)**
**14**	**Zihl (**1988**)**
**15**	**Kuester-Gruber et al. (**2020**)**
**16**	**de Jong et al. (**2016**)**
**17**	**Mohamad et al. (**2021**)**
**18**	**Lacey et al. (**2015**)**
**19**	**Mena-Garcia et al. (**2021**)**
**20**	**Woodhead et al. (**2013**)**
21	Crotty et al. (2018)
22	Rowe et al. (2017)
23	de Haan et al. (2016)
24	Levy-Bencheton et al. (2016)
25	de Haan et al. (2015b)
26	Larson (2015)
27	Jacquin-Courtois et al. (2013)
28	Hayes et al. (2012)
29	Mödden et al. (2012)
30	Lane et al. (2010)
31	Roth et al. (2009)
32	Bolognini et al. (2005)
33	Nelles et al. (2001)
34	Vasiliou (1990)
35	Zihl and von Cramon (1985)
36	Bergsma et al. (2017)
37	Elshout et al. (2016)
38	Bergsma et al. (2014)
39	Bergsma et al. (2012)
40	Gall and Sabel (2012)
41	Bergsma and van der Wildt (2010)
42	Keller and Lefin-Rank (2010)
43	Poggel et al. (2010)
44	Jobke et al. (2009)
45	Gall et al. (2008)
46	Poggel et al. (2008)
47	Mueller et al. (2007)
48	Reinhard et al. (2005)
49	Sabel et al. (2004)
50	Mueller et al. (2003)
51	Daibert-Nido et al. (2021)
52	Grasso et al. (2016)
53	Passamonti et al. (2009)
54	Plow et al. (2012b)
55	Plow et al. (2011)
56	Räty et al. (2021)
57	Bowers et al. (2008)
58	Selivanova et al. (2019)
59	Smaakjær et al. (2018)
60	Plow et al. (2012a)

Bolded references indicate a reference including a reading intervention

## Appendix B

**Table:**

1 = Yes 2 = No 3 = Don’t know	% positive score of item	1.Zihl	2.Hazelton	3.Pineda-Ortiz	4.Aimola	5.Schuett	6.Ong	7.Schuett	8.Kerkhoff	9.Schuett	10.Spitzyna	11.Ciuffreda	12.Zihl	13.Kerkhoff	14.Zihl
***Percentage positive scores of record***		**75**	**80**	**55**	**80**	**75**	**85**	**75**	**10**	**75**	**70**	**80**	**55**	**55**	**40**
*Introduction*															
1. Were the aims/objectives of the study clear?	93	1	1	1	1	1	1	1	1	1	1	1	1	1	1
*Methods*															
2. Was the study design appropriate for the stated aim(s)?	73	1	1	1	1	1	1	1	?	1	1	1	1	1	0
3. Was the sample size justified?	7	0	0	0	0	0	0	0	0	0	0	0	0	0	0
4. Was the target/reference population clearly defined? (Is it clear who the research was about?)	95	1	1	1	1	1	1	1	0	1	1	1	1	1	1
5. Was the sample frame taken from an appropriate population base so that it closely represented the target/reference population under investigation?	95	1	1	1	1	1	1	1	?	1	0	1	1	1	1
6. Was the selection process likely to select subjects/participants that were representative of the target/reference population under investigation?	55	1	?	1	1	1	1	?	?	?	?	1	?	?	?
7. Were measures undertaken to address and categorise non-responders?	35	1	1	0	1	0	1	0	0	0	0	0	0	0	0
8. Were the risk factor and outcome variables measured appropriate to the aims of the study?	82	1	1	?	1	1	1	1	?	1	1	1	1	1	1
9. Were the risk factor and outcome variables measured correctly using instruments/measurements that had been trialed, piloted or published previously?	42	0	1	0	0	1	0	1	0	1	1	?	?	0	?
10. Is it clear what was used to determined statistical significance and/or precision estimates? (e.g. p-values, confidence intervals)	68	1	1	0	0	1	1	1	0	1	1	1	1	0	1
11. Were the methods (including statistical methods) sufficiently described to enable them to be repeated?	73	1	1	0	1	1	1	1	0	1	1	1	0	1	0
*Results*															
12. Were the basic data adequately described?	90	1	0	1	1	1	1	1	0	1	1	1	1	1	1
13. Does the response rate raise concerns about non-response bias?	85	1	0	0	0	0	1	0	?	0	0	0	0	1	?
14. If appropriate, was information about non-responders described?	33	0	1	0	1	0	1	0	0	0	0	0	0	1	0
15. Were the results internally consistent?	83	1	1	?	1	1	1	1	?	1	1	1	1	1	1
16. Were the results presented for all the analyses described in the methods?	70	1	1	?	1	1	1	1	?	1	1	1	?	?	?
*Discussion*															
17. Were the authors’ discussions and conclusions justified by the results?	78	1	1	1	1	1	1	1	1	1	1	1	1	1	0
18. Were the limitations of the study discussed?	42	0	0	1	0	0	1	0	0	0	0	1	0	0	0
*Other*															
19. Were there any funding sources or conflicts of interest that may affect the author’s interpretation of the results?	80	0	0	0	0	0	0	0	1	0	0	0	0	0	0
20. Was ethical approval or consent of participants attained?	77	1	1	1	1	0	1	1	?	1	1	1	?	?	?

1 = Yes 2 = No 3 = Don’t know	15.Kuester-Gruber	16.de Jong	17.Mohamad	18.Lacey	19.Mena-Garcia	20.Woodhead	21.Crotty	22.Rowe	23.de Haan	24.Levy-Bencheton	25.de Haan	26.Larson	27.Jacquin-Courtois	28.Hayes	29.Mödden
***Percentage positive scores***	**95**	**65**	**65**	**55**	**85**	**70**	**95**	**80**	**90**	**75**	**90**	**40**	**70**	**60**	**75**
*Introduction*															
1. Were the aims/objectives of the study clear?	1	1	1	1	1	1	1	1	1	1	1	0	1	1	1
*Methods*															
2. Was the study design appropriate for the stated aim(s)?	1	1	1	0	1	1	1	1	1	1	1	?	1	0	1
3. Was the sample size justified?	0	0	1	0	0	0	1	0	0	0	1	0	0	0	0
4. Was the target/reference population clearly defined? (Is it clear who the research was about?)	1	1	1	1	1	1	1	1	1	1	1	1	1	1	1
5. Was the sample frame taken from an appropriate population base so that it closely represented the target/reference population under investigation?	1	1	1	1	1	1	1	1	1	1	1	1	1	1	1
6. Was the selection process likely to select subjects/participants that were representative of the target/reference population under investigation?	1	1	1	1	1	?	1	1	1	1	1	1	1	1	1
7. Were measures undertaken to address and categorise non-responders?	1	0	0	0	0		1	1	1	0	1	0	0	0	0
8. Were the risk factor and outcome variables measured appropriate to the aims of the study?	1	1	1	1	1	1	1	1	1	1	1	0	1	1	?
9. Were the risk factor and outcome variables measured correctly using instruments/measurements that had been trialed, piloted or published previously?	1	1	1	0	1	0	1	1	1	0	1	?	0	1	0
10. Is it clear what was used to determined statistical significance and/or precision estimates? (e.g. p-values, confidence intervals)	1	1	0	0	1	1	1	1	1	1	1	0	1	0	1
11. Were the methods (including statistical methods) sufficiently described to enable them to be repeated?	1	0	0	0	1	1	1	1	1	1	1	0	1	1	0
*Results*															
12. Were the basic data adequately described?	1	1	1	1	1	1	1	1	1	1	1	1	1	1	1
13. Does the response rate raise concerns about non-response bias?	0	0	0	0	0	0	0	0	0	0	1	0	0	0	0
14. If appropriate, was information about non-responders described?	1	0	0	0	1	0	1	1	0	0	0	0	0	1	1
15. Were the results internally consistent?	1	1	1	1	1	1	1	?	1	1	1	1	1	?	1
16. Were the results presented for all the analyses described in the methods?	1	?	?	?	1	1	1	0	1	1	1	?	1	0	1
*Discussion*															
17. Were the authors’ discussions and conclusions justified by the results?	1	0	1	1	1	1	1	?	1	1	1	1	1	?	1
18. Were the limitations of the study discussed?	1	0	0	0	1	0	1	1	1	0	1	1	0	1	1
*Other*															
19. Were there any funding sources or conflicts of interest that may affect the author’s interpretation of the results?	0	0	0	0	1	0	1	0	0	0	0	?	?	1	0
20. Was ethical approval or consent of participants attained?	1	1	?	1	1	1	1	1	1	1	1	?	1	1	1

1 = Yes 2 = No 3 = Don’t know	30.Lane	31.Roth	32.Bolognini	33.Nelles	34.Vasiliou	35.Zihl	36.Bergsma	37.Elshout	38.Bergsma	39.Bergsma	40.Gall	41.Bergsma	42.Keller	43.Poggel	44.Jobke
***Percentage positive scores***	**70**	**65**	**55**	**40**	**15**	**55**	**70**	**75**	**75**	**50**	**65**	**50**	**80**	**70**	**60**
*Introduction*															
1. Were the aims/objectives of the study clear?	1	1	1	0	?	1	1	0	1	1	1	1	1	1	1
*Methods*															
2. Was the study design appropriate for the stated aim(s)?	1	1	0	0	0	1	1	1	0	0	1	0	1	1	1
3. Was the sample size justified?	0	0	0	0	0	0	0	0	0	0	0	0	0	0	0
4. Was the target/reference population clearly defined? (Is it clear who the research was about?)	1	1	1	0	1	1	1	1	1	1	1	0	1	1	1
5. Was the sample frame taken from an appropriate population base so that it closely represented the target/reference population under investigation?	0	1	1	1	1	1	1	1	1	1	1	1	1	1	1
6. Was the selection process likely to select subjects/participants that were representative of the target/reference population under investigation?	0	?	0	?	?	?	1	1	?	1	?	0	1	?	?
7. Were measures undertaken to address and categorise non-responders?	1	1	0	0	0	0	1	0	1	0	0	0	0	0	0
8. Were the risk factor and outcome variables measured appropriate to the aims of the study?	1	1	0	?	?	1	1	1	?	1	1	1	1	1	1
9. Were the risk factor and outcome variables measured correctly using instruments/measurements that had been trialed, piloted or published previously?	0	1	?	0	?	?	0	0	1	0	1	0	0	0	1
10. Is it clear what was used to determined statistical significance and/or precision estimates? (e.g. p-values, confidence intervals)	1	0	1	0	0	1	0	1	1	0	0	0	1	1	1
11. Were the methods (including statistical methods) sufficiently described to enable them to be repeated?	1	1	1	1	0	0	0	1	1	0	1	1	1	1	1
*Results*															
12. Were the basic data adequately described?	1	0	1	1	0	1	1	1	1	1	1	1	1	1	1
13. Does the response rate raise concerns about non-response bias?	0	0	0	0	0	0	0	0	0	0	0	1	0	0	1
14. If appropriate, was information about non-responders described?	0	0	0	0	0	0	1	1	0	0	0	1	0	0	0
15. Were the results internally consistent?	1	?	1	1	?	1	0	1	1	0	0	1	1	1	1
16. Were the results presented for all the analyses described in the methods?	1	1	?	1	?	0	1	1	1	?	1	?	1	1	1
*Discussion*															
17. Were the authors’ discussions and conclusions justified by the results?	1	1	1	?	0	1	1	1	1	0	1	0	1	1	?
18. Were the limitations of the study discussed?	0	0	0	0	0	0	0	0	1	1	1	1	1	0	0
*Other*															
19. Were there any funding sources or conflicts of interest that may affect the author’s interpretation of the results?	0	0	0	0	1	0	0	0	0	0	0	0	0	0	?
20. Was ethical approval or consent of participants attained?	1	1	1	1	?	?	1	1	1	1	?	1	1	1	1

1 = Yes 2 = No 3 = Don’t know	45.Gall	46.Poggel	47.Mueller	48.Reinhard	49.Sabel	50.Mueller	51.Daibert-Nido	52.Grasso	53.Passamonti	54.Plow	55.Plow	56.Räty	57.Bowers	58.Selivanova	59.Smaakjaer
***Percentage positive scores***	**75**	**70**	**50**	**60**	**75**	**50**	**90**	**60**	**70**	**70**	**60**	**55**	**75**	**95**	**75**
*Introduction*															
1. Were the aims/objectives of the study clear?	1	1	1	1	1	1	1	1	1	1	1	1	1	1	1
*Methods*															
2. Was the study design appropriate for the stated aim(s)?	1	1	0	0	1	0	1	0	1	1	0	0	1	1	1
3. Was the sample size justified?	0	0		0	0	0	0	0	0	0	0	0	0	0	1
4. Was the target/reference population clearly defined? (Is it clear who the research was about?)	1	1	1	1	1	1	1	1	1	1	1	1	1	1	1
5. Was the sample frame taken from an appropriate population base so that it closely represented the target/reference population under investigation?	1	1	1	1	1	1	1	1	1	1	1	1	1	1	1
6. Was the selection process likely to select subjects/participants that were representative of the target/reference population under investigation?	?	1	1	1	1	?	1	?	?	1	?	0	?	1	1
7. Were measures undertaken to address and categorise non-responders?	1	0	1	1	1	1	1	0	0	0	0	0	0	1	0
8. Were the risk factor and outcome variables measured appropriate to the aims of the study?	1	1	?	1	1	?	1	1	1	1	1	0	1	1	1
9. Were the risk factor and outcome variables measured correctly using instruments/measurements that had been trialed, piloted or published previously?	1	0	0	0	0	0	1	0	0	1	1	?	1	1	0
10. Is it clear what was used to determined statistical significance and/or precision estimates? (e.g. p-values, confidence intervals)	1	1	1	0	1	1	0	1	1	1	0	1	0	1	1
11. Were the methods (including statistical methods) sufficiently described to enable them to be repeated?	1	1	0	1	1	0	1	1	1	1	1	1	1	1	1
*Results*															
12. Were the basic data adequately described?	1	1	1	1	1	1	1	1	1	0	1	1	1	1	0
13. Does the response rate raise concerns about non-response bias?	0	0	0	1	0	1	0	0	0	0	0	0	0	0	1
14. If appropriate, was information about non-responders described?	1	0	0	1	0	0	1	0	0	1	0	0	0	1	0
15. Were the results internally consistent?	1	1	1	1	1	1	1	1	1	?	1	1	1	1	1
16. Were the results presented for all the analyses described in the methods?	1	1	1	?	1	1	1	1	1	1	?	?	1	1	1
*Discussion*															
17. Were the authors’ discussions and conclusions justified by the results?	0	1	?	1	1	1	1	?	1	0	1	1	1	1	1
18. Were the limitations of the study discussed?	1	0	0	1	0	0	1	0	0	0	1	0	1	1	1
*Other*															
19. Were there any funding sources or conflicts of interest that may affect the author’s interpretation of the results?	1	1	1	?	1	0	0	0	0	0	1	0	0	0	0
20. Was ethical approval or consent of participants attained?	?	1	?	?	1	?	1	1	1	1	1	1	1	1	1

1 = Yes 2 = No 3 = Don’t know	60.Plow
***Percentage positive scores***	**90**
*Introduction*	
1. Were the aims/objectives of the study clear?	1
*Methods*	
2. Was the study design appropriate for the stated aim(s)?	1
3. Was the sample size justified?	0
4. Was the target/reference population clearly defined? (Is it clear who the research was about?)	1
5. Was the sample frame taken from an appropriate population base so that it closely represented the target/reference population under investigation?	1
6. Was the selection process likely to select subjects/participants that were representative of the target/reference population under investigation?	1
7. Were measures undertaken to address and categorise non-responders?	1
8. Were the risk factor and outcome variables measured appropriate to the aims of the study?	1
9. Were the risk factor and outcome variables measured correctly using instruments/measurements that had been trialed, piloted or published previously?	1
10. Is it clear what was used to determined statistical significance and/or precision estimates? (e.g. p-values, confidence intervals)	1
11. Were the methods (including statistical methods) sufficiently described to enable them to be repeated?	1
*Results*	
12. Were the basic data adequately described?	1
13. Does the response rate raise concerns about non-response bias?	0
14. If appropriate, was information about non-responders described?	1
15. Were the results internally consistent?	1
16. Were the results presented for all the analyses described in the methods?	1
*Discussion*	
17. Were the authors’ discussions and conclusions justified by the results?	1
18. Were the limitations of the study discussed?	1
*Other*	
19. Were there any funding sources or conflicts of interest that may affect the author’s interpretation of the results?	1
20. Was ethical approval or consent of participants attained?	1

## Abbreviations

AXIS: Appraisal tool for cross-sectional studies
FU: Follow-up
GAS: Goal attainment score
HH: Homonymous hemianopia
HVFD: Homonymous visual field defect
iHH: Incomplete homonymous hemianopia
iQA: Incomplete homonymous quadrantanopia
IReST: International Reading Speed Texts
IVI: Impact of visual impairment questionnaire
iwH: Individual with homonymous visual field defect
LogRAD: Radner reading acuity score
MNREAD: Minnesota Low-Vision Reading Test
NEI-VFQ-25: 25-Item National Eye Institute Visual Function Questionnaire
NIBS: Non-Invasive Brain Stimulation
OI: Other intervention
PRISMA-P: Preferred Reporting Items for Systematic review and Meta-Analysis Protocol
QA: Homonymous quadrantanopia
RCT: Randomised Controlled Trial
RI: Reading intervention
UK: United Kingdom
USA: United States of America
VA LV VFQ-48: Veterans Affair Low-Vision Visual Functioning Questionnaire
VIQ: Visual Impairments Questionnaire
VSRT: Pepper Visual Skills for Reading Test
